# Fitting and Interpreting Occupancy Models

**DOI:** 10.1371/journal.pone.0052015

**Published:** 2013-01-10

**Authors:** Alan H. Welsh, David B. Lindenmayer, Christine F. Donnelly

**Affiliations:** 1 Centre for Mathematics and its Applications, The Australian National University, Canberra, Australia; 2 Fenner School of Environment and Society, The Australian National University, Canberra, Australia; Utah State University, United States of America

## Abstract

We show that occupancy models are more difficult to fit than is generally appreciated because the estimating equations often have multiple solutions, including boundary estimates which produce fitted probabilities of zero or one. The estimates are unstable when the data are sparse, making them difficult to interpret, and, even in ideal situations, highly variable. As a consequence, making accurate inference is difficult. When abundance varies over sites (which is the general rule in ecology because we expect spatial variance in abundance) and detection depends on abundance, the standard analysis suffers bias (attenuation in detection, biased estimates of occupancy and potentially finding misleading relationships between occupancy and other covariates), asymmetric sampling distributions, and slow convergence of the sampling distributions to normality. The key result of this paper is that the biases are of similar magnitude to those obtained when we ignore non-detection entirely. The fact that abundance is subject to detection error and hence is not directly observable, means that we cannot tell when bias is present (or, equivalently, how large it is) and we cannot adjust for it. This implies that we cannot tell which fit is better: the fit from the occupancy model or the fit ignoring the possibility of detection error. Therefore trying to adjust occupancy models for non-detection can be as misleading as ignoring non-detection completely. Ignoring non-detection can actually be better than trying to adjust for it.

## Introduction

Detection error is a widely acknowledged problem in the collection of ecological data. Detection error complicates estimation and modelling because it is difficult to separate the ecological and the detection processes in the analysis. Intuitively, it should be easier to detect and adjust for whole species (occupancy) than for all the individuals of a species (abundance). For this reason, occupancy, which is a quantity of ecological interest in its own right, is sometimes studied as a surrogate for abundance [1]. Occupancy modelling (described in [1]) is based on making multiple visits to some or all of the sample sites in a study to collect species detection data which are used to model, and then adjust for, the detection process. There is a growing literature on occupancy modelling and many apparently successful applications. The methodology seems to be widely viewed as having achieved the status of a “gold standard” for analysing ecological data which are subject to detection error.

This paper is motivated by our fitting the occupancy models of [1] to some data from a major study in South-eastern Australia [2], [3] which is described in the Analysis and results section below. The original purpose of the study was to investigate changing patterns in the abundance of different species in remnant mature woodland patches surrounded by maturing Radiata pine (*Pinus radiata*). However, reviewers of that work directed us to fit occupancy models to our data to model detection and occupancy instead. This advice is consistent with the general recommendation of [1] that we should study changes in occupancy instead of abundance. For the purposes of this paper, we take the view that occupancy models are defined by [1]. Although we have data for several species over several seasons, we started to explore the use of occupancy models in the simplest case by considering single-species, single-season models in detail. We fully recognise that some more complicated models are included in [1] and others have appeared in the literature since then. We feel strongly that it is important to study the simplest cases first so that we can build up experience and develop our intuition. This means that this paper is not intended to be the final word on using occupancy models to analyse our full set of data. It is rather a methodological investigation of the properties of the single-species, single-season occupancy model in simple situations.

**Figure 1:**
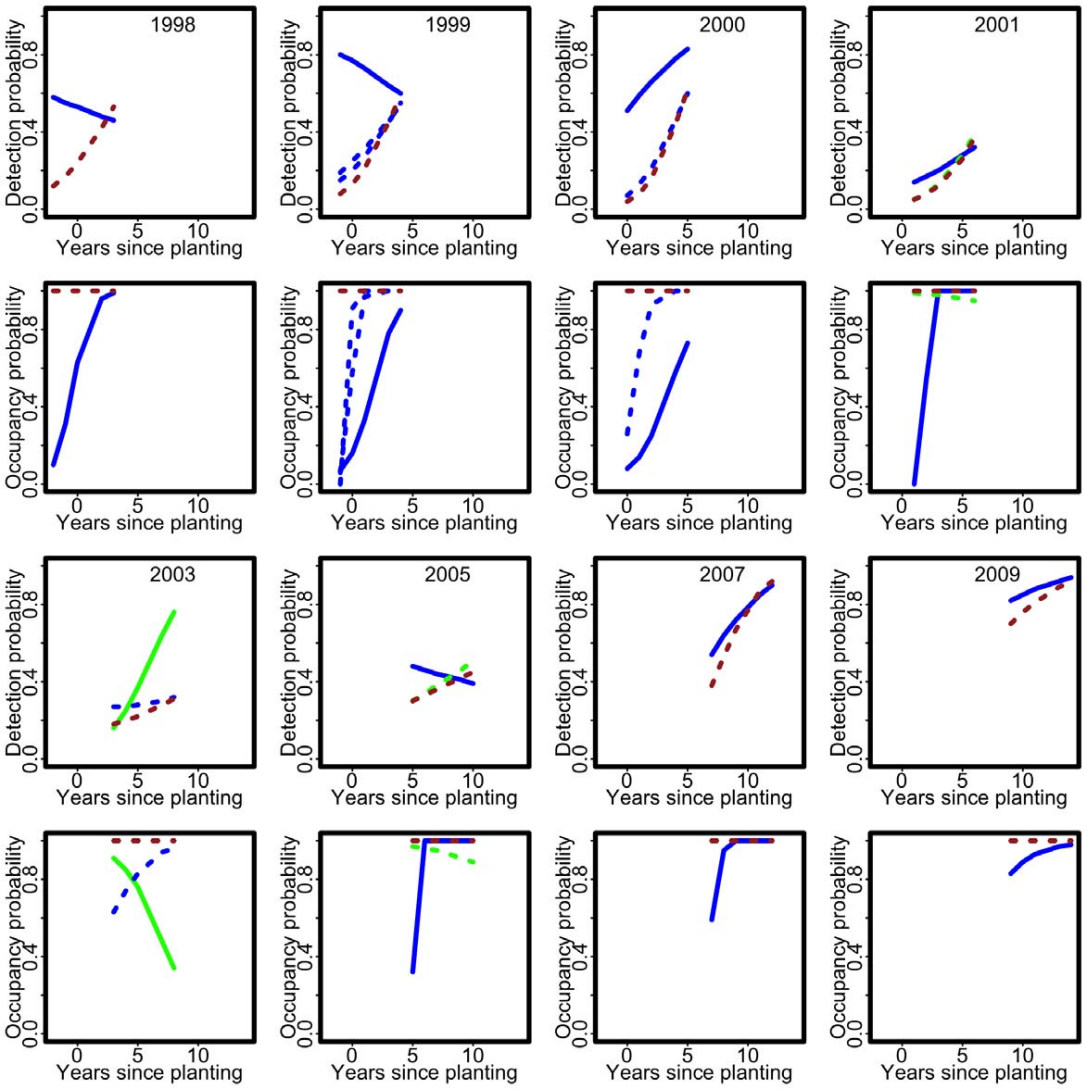
Attenuation in detection and its consequences for occupancy when detection depends on abundance. The first panel shows the distributions of the detection probabilities (with means represented by a circle) for each value of years since planting. The solid blue curve is the fitted logistic detection component when 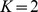 and the dashed blue curve is the fitted constant detection probability when 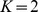. The solid green curve is the fitted logistic detection component when 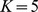 and the dashed green curve is the fitted constant detection probability when 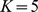. The solid brown curve is the fitted logistic detection component and the dashed brown curve is the fitted constant detection probability when 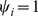 (and 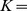
either 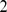 or 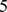). The pink and orange dashed line represents 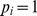 (i.e. ignoring non-detection). The second panel shows the corresponding fitted logistic occupancy probabilities in the same pattern and colour combinations. Note that orange dashed curve (slightly below the dashed blue curve) is the fitted logistic detection component when 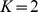 and the pink dashed curve (which coincides with the dashed green curve) is the fitted logistic detection component when 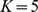.

The results of fitting occupancy models to our data (reported in the Analysis and results section) raise interesting questions about the use and interpretation of the methodology. These include: How often do we obtain multiple solutions and boundary estimates (probabilities of zero or one) from the estimating equations? Can we interpret both the relatively consistent pattern we find in one species and the lack of pattern in the other? What can we say about the uncertainty (sampling variability) in the estimates and how does this affect our interpretation of them? Does the modelled pattern of changes in detection within patches with the growth of the surrounding forest make sense? Does this change in detection have any effect on the relationship between occupancy and other covariates? A second, slightly more abstract motivation for our study is to address the question: Is adjustment for non-detection always worthwhile? The second panel of Figure 1 below is a version of the conceptual Figure 2.3 from [1] for a particular situation we consider. It will be discussed in more detail later but for now note that the solid black line shows a true (constant) relationship between occupancy and years since planting and the dashed orange and pink curves show apparent relationships when we ignore detection. The main reason for fitting occupancy models is to try to get much closer to the true relationship than the dashed orange and pink curves. Under ideal conditions, occupancy models do make a good adjustment, but is this always the case? These questions are important to ecology because answering them gets to the question of the value of occupancy modelling itself.

**Figure 2:**
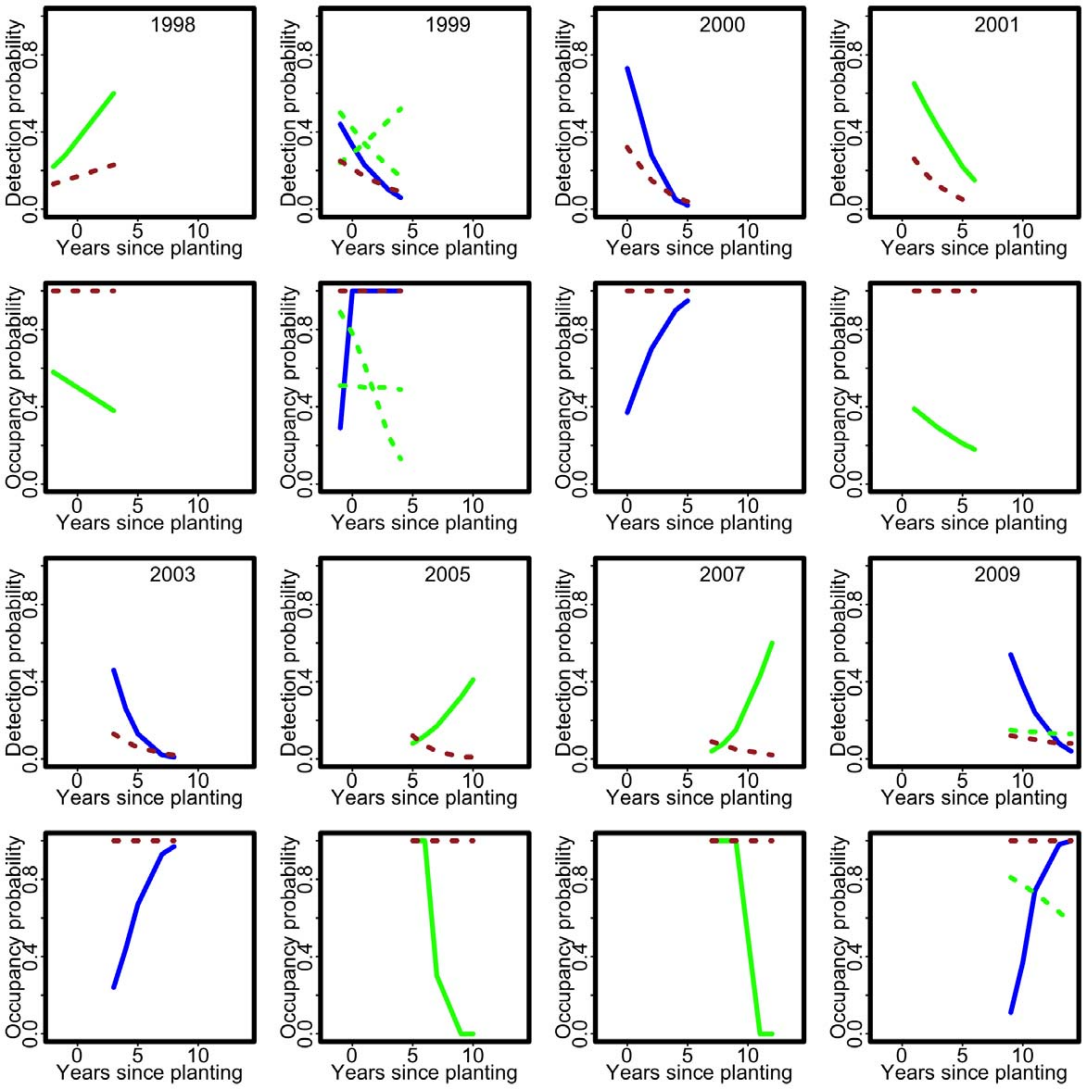
Fitted single-species, single-season detection and occupancy probabilities for the Brown Thornbill for 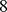
 **separate surveys in the Nanangroe Study.** The first and second rows show the fitted detection and occupancy probabilities for the first four surveys (1998–2001) and the third and fourth rows show the fitted detection and occupancy probabilities for the last four surveys (2003–2009). In each panel, the fitted probabilities with the highest log-likelihood are shown as a solid curve and the fitted probabilities corresponding to other solutions of the log-likelihood estimating equations are shown as dashed curves. Fitted models with increasing occupancy are shown in blue and those with decreasing occupancy in green. The fitted detection probabilities when 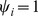 are shown in brown.

In this paper, we investigate the above questions, and illustrate and explain them using both simulation, and theoretical calculation. We place occupancy modelling in the wider context of nonlinear measurement error models to enhance our understanding of our results and to enable us to anticipate what may happen with other approaches to the same problem. We show that 1) the maximum likelihood estimating equations have multiple solutions, including some which produce fitted probabilities of zero or one; 2) that fitting the model to sparse data produces unstable fitted probabilities, including probabilities equal to one; 3) that for realistic survey effort, the fitted probabilities are highly variable, making inference and interpretation difficult; and, 4) when the detection process depends on abundance, the bias in the fitted probabilities can be of similar magnitude to the bias when the detection process is ignored, and this is very difficult to overcome. Point 4) is shown by the blue and green curves in the second panel in Figure 1 which are not very different from the unadjusted pink and orange curves. This is the key result of this paper as it undermines the rationale for occupancy modelling. It shows that when detection depends on abundance, ignoring non-detection can actually be better than trying to adjust for it, so the extra data collection and modelling effort to try to adjust for non-detection is simply not worthwhile. A reviewer of the original version of this paper expressed the opinion that 1)–3) are well-known to practitioners. We find this difficult to evaluate; they are not mentioned in [1] or, as far as we know, in the methodological literature where we would expect at least 1) and 3) to be discussed. We find 2) quite surprising as sparse data should lead only to zero or small fitted probabilities, whereas in fact it can lead to fitted values of both zero and one.

In practical terms, when investigating relationships between occupancy and other variables measured in a study, we can find spurious relationships, make these relationships seem stronger or weaker than they are, or fail to find real relationships. In addition, for any given set of data, because we cannot observe abundance and the distribution of abundance is not identifiable, we have no way of knowing whether anything we find out about relationships is correct or not. In this situation, the occupancy model and the much criticised strategy of ignoring the detection process both give answers which can be misleading to a similar, unknown extent.

Generally, statistical methods apply in quite specific situations, under quite specific conditions. If the conditions are either not made explicit or are made explicit but then largely ignored, the methods start to be treated as being much more widely applicable than they are. It is important to be honest about the limitations of procedures. Modelling and adjusting for non-detection is very difficult, and simple solutions are mostly applicable only in limited circumstances. In particular, occupancy modelling is not always applicable; it should not be used indiscriminately or recommended as a “gold standard” for adjusting models for site occupancy for the effect of non-detection.

## Analysis and Results

The overall purpose of this study is to describe the performance of occupancy models in some realistic situations. Occupancy models are usually fitted to data (i.e. the unknown parameters are estimated) by maximising the likelihood, so we investigate the existence of multiple solutions to the maximum likelihood estimating equations, the occurrence of fitted boundary probabilities (i.e. estimated probabilities of zero or one), the effect of sparse data, and the effect of abundance. Our analysis includes the empirical analysis of a real data set, numerical simulations under particular settings and theoretical calculations. We also compare the theoretical calculations and simulation results for occupancy models with those obtained when we ignore the possibility of non-detection.

### Ethics statement

The research was carried out in accordance with the requirements of permit F.ES.04.10 issued by the Animal Experimentation Ethics Committee of The Australian National University. We also obtained a scientific research license issued by the New South Wales Parks and Wildlife Service (no. 13174). The relevant permissions for State Forests were given by staff from the Tumut Office of State Forests of New South Wales. All native animal species and native woodland vegetation, including endangered birds and plants, are protected in Australia. Our studies were observational investigations and no plants or animals were harmed in any way.

### Empirical analysis

We fitted occupancy models to data on the Brown Thornbill (*Acanthiza pusilla*) and the Yellow-rumped Thornbill (*Acanthiza chrysorrhoa*) collected from 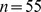 sites as part of the Nanangroe Study in South-eastern Australia [2], [3]. The sites are remnant mature woodland patches surrounded by a maturing Radiata pine plantation; they can be grouped into 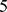 cohorts corresponding to the different years in which the surrounding Radiata pine trees were planted. Since the start of the study, surveys have been conducted to determine how biota in the woodland patches changes as the plantation matures. In this paper, we consider data gathered in 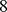 surveys conducted between 1998 and 2009. In each of the 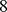 surveys, each of the 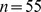 patches was visited on 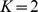 different days by different observers who recorded whether they detected the species or not. Each observer made their observation in a patch from 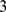 points 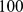 m apart on a transect in the patch and the species is detected if it is heard from at least one of the 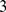 points during a visit. Making only 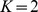 visits to each patch in each survey is considered a low number of repeat visits and 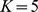 or more visits is often recommended [4]. However, these are historical data (from a study which was not designed specifically to collect occupancy data) and we cannot change what was done previously. Moreover, even if the study was redesigned, it is beyond our capacity to increase the number of visits. As we will see, the points we are making are not all resolved simply by increasing the number of visits to each patch.

To model the data for one species in a single survey, let 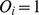 if patch 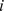 is occupied by the species and 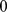 otherwise, and let 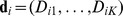 denote the “detection history” at site 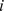, where 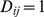 if the species is detected at patch 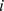 in visit 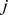 and 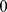 otherwise. Then we assume the occupancy model
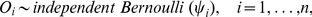
(1)

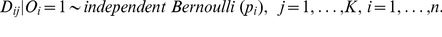
(2)We can obtain a slightly more general version of the occupancy model by replacing 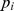 by 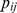, and we can let 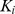 vary with patch, but the present, simpler version is adequate for our data. Following [1], we assume that for a given survey (i) occupancy does not change over the visits and (ii) there are no false detections, so there is no measurement error when the patch is unoccupied (i.e. 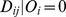 equals zero with probability one).

Since we are interested in describing changes in occupancy as the Radiata pine stands surrounding the woodland patches mature, we let both the occupancy and detection probabilities be a function of 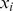, the years since planting of the Radiata pine stands. We adopt the usual logistic regression formulation [1]

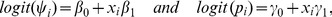
(3)where 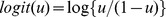 and 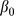, 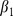, 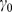, 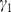 are unknown parameters. Let 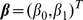, 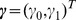 and 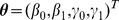. We will take it as understood that the occupancy model (1)–(2) includes (3), unless otherwise stated. When 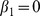, the model has constant occupancy and, when 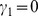, constant detection. We will at times fit the occupancy model with constant occupancy and/or constant detection as particular cases of the occupancy model.

To fit the occupancy model (1)–(3), we used the function **vglm** from the VGAM package [5] in R. VGAM is a very high quality, flexible, general package which fits a wide variety of models. It is not our purpose to critique different software so we simply chose a very reliable implementation.

We first fitted the occupancy model separately to the data for the two species from the first survey. These results encouraged us to fit the model to more data so, to take advantage of the data we have, we then fitted the logistic occupancy model separately to each species and each survey. As we explained in the Introduction, this is not intended to be a definitive analysis of our data, but rather a preliminary exploration of how the single-species, single-season occupancy model performs over several data sets. The fact that our data sets are closely related should make it easier to identify unusual or inconsistent results. Indeed, our analysis highlights a number of interesting points and motivates the further investigations reported in this paper.

### Empirical results

**Figure 3:**
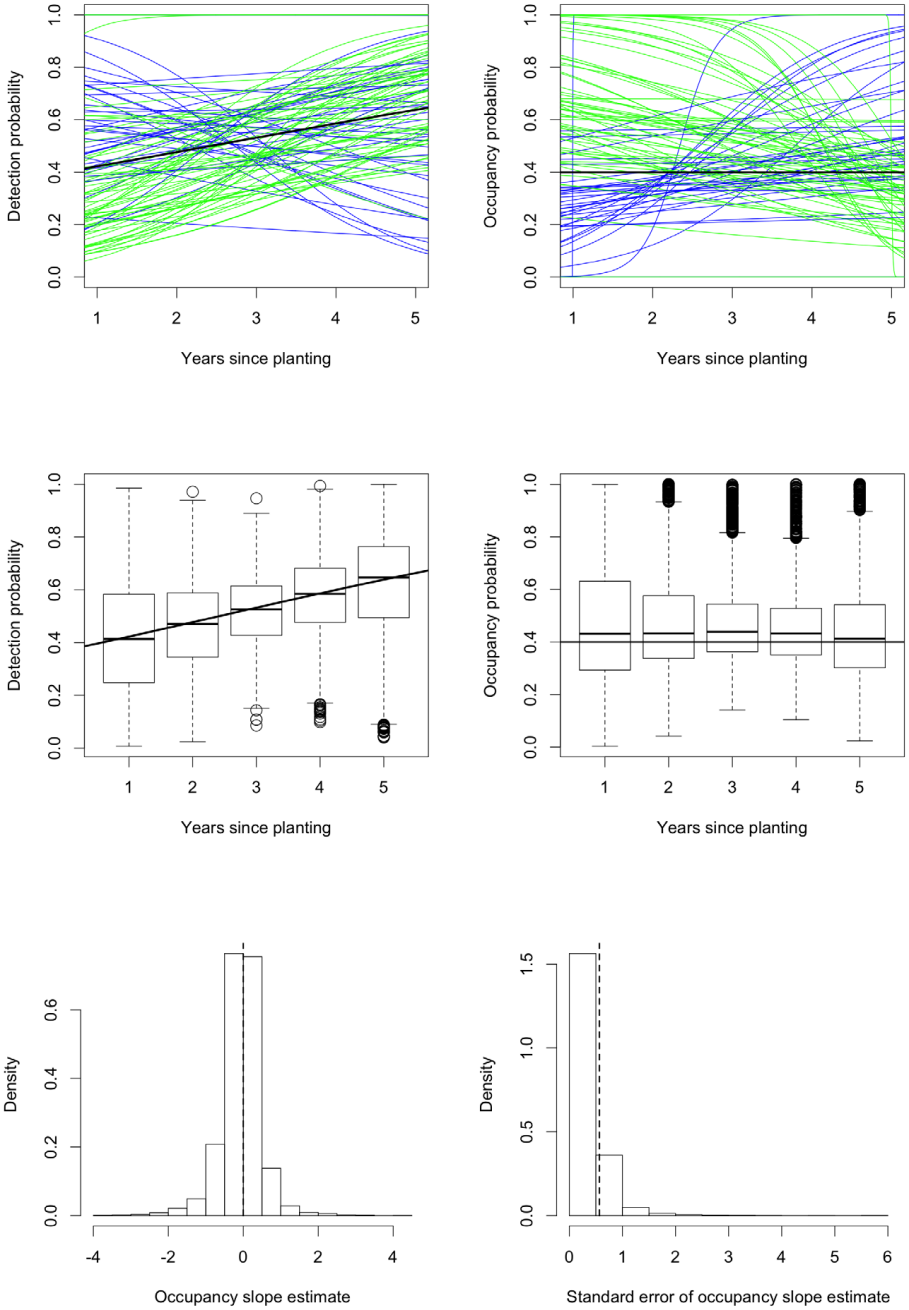
Fitted single-species, single-season detection and occupancy probabilities for the Yellow-rumped Thornbill for 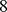
 **separate surveys in the Nanangroe Study.** The first and second rows show the fitted detection and occupancy probabilities for the first four surveys (1998–2001) and the third and fourth rows show the fitted detection and occupancy probabilities for the last four surveys (2003–2009). In each panel, the fitted probabilities with the highest log-likelihood are shown as a solid curve and the fitted probabilities corresponding to other solutions of the log-likelihood estimating equations are shown as dashed curves. Fitted models with increasing occupancy are shown in blue and those with decreasing occupancy in green. The fitted detection probabilities when 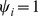 are shown in brown.

The fitted single-species, single-season occupancy models for the Brown Thornbill and the Yellow-rumped Thornbill in the 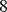 surveys from the Nanangroe Study are shown as solid curves in Figures 2–3. For both the Brown Thornbill and the Yellow-rumped Thornbill, there are extreme fitted probabilities of zero and one, sometimes in the same survey. For the Brown Thornbill, except in 2003, occupancy tends to increase with years since planting, but for the Yellow-rumped Thornbill there is no consistent pattern with increases in some surveys and decreases in others. Moreover, the fitted occupancy probabilities oscillate wildly, showing occupancies of both zero and one. In the 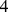 surveys from 2003–2009, there are only 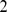 sites in which the Yellow-rumped Thornbill is detected on both visits, and in 2005 and 2007 there are two cohorts (corresponding to different years since planting) with no detections at all, so the Yellow-rumped Thornbill data are sparse.

We searched for other solutions to the maximum likelihood estimating equations by varying the starting values used in the numerical algorithm. We show the fitted probabilities of occupancy and detection corresponding to the other solutions we have been able to find for the surveys in the Nanangroe Study as dashed curves in Figures 2–3. The figures show that there are usually multiple different solutions to the maximum likelihood estimating equations so finding the maximum likelihood estimates is not as straightforward as simply solving the estimating equations. It is interesting that the multiple solutions are often quite similar, but this is not always the case (e.g. in the 2003 Brown Thornbill and the 1999 and 2009 Yellow-rumped Thornbill surveys). The function vglm with its default settings usually finds the maximum likelihood estimate; the exception is the 2001 Brown Thornbill survey where it finds the second solution and it is difficult to find the first solution without an extensive search using multiple starting values.

We could have pooled the data from the different surveys and fitted a single model to all 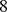 surveys. This would correspond to an 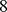-fold increase in the number of sites (ignoring any possible dependence) without resolving the issues. (We do consider the effect of increasing the sample size in a simulation below.) Actually, a more useful change would be to increase the number of parameters by making years since planting a factor so that we do not impose the linear logistic constraints as we have done. In this case, within each survey the effect of years since planting would be estimated from 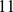 sites which is rather low.

### Multiple solutions and boundary estimates: Theoretical calculations

We investigate the properties of fitted occupancy models through the log-likelihood for the unknown parameters 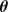 and the corresponding maximum likelihood estimating equations 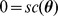, where the score function 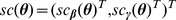 is obtained by differentiating the log-likelihood with respect to the unknown parameters 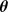.

The estimating equations are nonlinear in 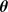 so explicit solutions are not available and we need to use numerical methods to obtain their solutions. We again chose to use the function **vglm** from the VGAM package [5] in R. We also used the function **nleqslv** from the nleqslv package [6] in R to search for multiple solutions to the estimating equations and to solve the expected estimating equations (see below).

### Multiple solutions and boundary estimates: Theoretical results

In general, suppose that we observe vectors of covariates 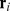 and 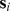 which we want to relate to the occupancy and the detection probability, respectively, using the logistic models
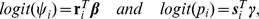
where 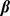 and 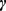 are unknown vector parameters of the same dimension as  and 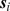, respectively. For the models fitted to the Nanangroe Study data, we had 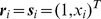, 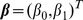 and 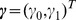. Let 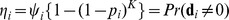, where 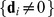 is the event that at least one component of 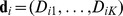 is nonzero. Then the log-likelihood is
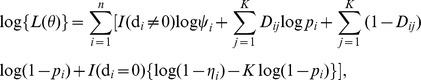
where 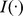 is the indicator function. The maximum likelihood estimate 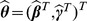 of 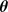 satisfies the estimating equations 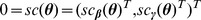, where the score functions (obtained by differentiating the log-likelihood with respect to the unknown parameters) are
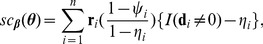
(4)

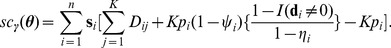
(5)Approximate standard errors can be obtained as usual (see for example [6]) by taking the square root of the diagonal elements of the inverse Fisher information matrix (see Information S1) evaluated at 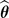.

Regardless of the data or the underlying data generating process, the estimating equation based on (4) always has a solution at 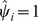. This solution corresponds to treating all sites as occupied and modelling any variability in the data as being due to the detection process. There will usually also be other solutions with some or all 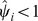. When this occurs, we need to compute the likelihood of each solution and check which maximises the likelihood to find the maximum likelihood estimate. It is also possible to have 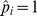 as a solution to the estimating equation based on (5) although, unlike 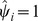, it is not always a solution. The solution 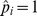 corresponds to treating detection as perfect and modelling any variability as due to the occupancy process. Simply imposing 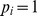 and solving the estimating equation based on (4) corresponds to ignoring the possibility of non-detection.

Boundary solutions such as 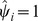 are a problem for logistic occupancy models because it means that at least one of the components of 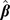 is set to infinity. The other components of 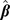 can take any value so there are uncountably many solutions to the estimating equations. In practice, the estimate does not achieve infinity but becomes large and, once it becomes large, the Hessian matrix, and hence the Fisher information matrix, becomes singular with zero blocks (see Information S1) so the algorithm stops. This happens for a wide range of large parameter values with a corresponding wide range of large standard errors, so the values of 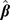 are ambiguous even though the fitted probabilities 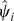 are well defined. A similar result applies for zero probabilities, where one of the components of 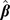 is set to negative infinity, and to cases where some of the fitted probabilities equal one or zero. We can illustrate this using a simplified example based on one of the simulated data sets we generated. (Details of the simulation are given in the next subsection.) For occupancy, we obtained estimates (on the logistic scale) of roughly 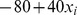, giving 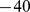, 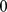, 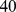, 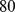, 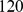 across the range of 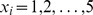 values. These values transform back to the probability scale (inverse logistic transformation) as 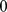, 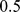, 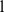, 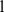, 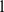. The standard errors for the intercept and slope are huge (they can be of the order of 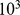 or greater) and so give a huge value for the standard error of the fitted value 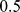. We get essentially the same fitted values on the probability scale if we multiply the intercept and slope estimates by the same multiple greater than one. i.e. we get essentially the same fitted values on the probability scale from 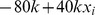 for any value of 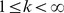. However, the standard errors also change with 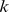 and translate to different standard errors for the fitted values. The intercept and slope parameter estimates (equivalently, the choice of 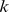) are determined by the software we use so are therefore quite arbitrary. These kinds of results, called abnormal convergence by [7], also occur in fitting ordinary binary regression models (including logistic models) if the data are sparse and some of the counts are all presences or all absences. We prefer the more descriptive terms boundary and interior to describe abnormal and normal convergence, estimates or fits, respectively. The main practical consequence is that with boundary estimates it is sensible to report and discuss fitted probabilities and log-likelihoods rather than parameter estimates and standard errors.

### Multiple solutions and boundary estimates: Simulation

In all our simulation studies, we used a setting based on an idealised single survey for a single species in the Nanangroe Study. We used 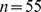 sites, 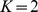 visits and set the years since planting the surrounding Radiata pine 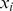 equal to 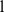 for 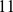 sites, 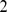 for 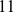 sites, and so on, up to 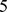 for 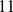 sites. Later, when we need to explore the effects of increasing survey effort, we also considered 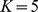 visits and 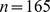 sites (scaling up by a factor of 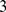). The total number of visits in a survey is 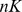, which for these four cases is 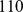, 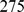, 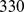 and 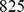, so all except the first case are well beyond what can realistically be implemented.

To simulate an ideal situation (under which everything should work very well and hence provide a baseline for later comparison), we set the occupancy probability 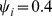 and detection probability 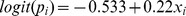 (so 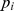 is approximately 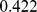, 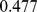, 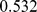, 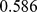 and 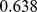). The results of [8] and [9] on how detection probability varies with foliage density imply that we should expect detection probability to decrease with years since planting (see the Discussion of the Nanangroe Study below). Our choice to allow the opposite was based on 1) the empirical results obtained by fitting the occupancy model to the Brown Thornbill data which show that the fitted detection probability can increase or decrease but overall tends to increase with years since planting and 2) our desire to make it easier to compare the results we obtain here with those from later simulation results in which it is natural to allow detection to increase with abundance. For each sample, we generated single species detection data from this occupancy model with constant occupancy and a logistic detection component and fitted the occupancy model (1)–(3) with 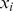 as the covariate. Since the model we are fitting contains the data generating model, we are fitting a correct model. In simulations with binary data, it is common to find that the estimation method does not converge in a small number of samples. This occurs more frequently in small samples than large samples and with misspecified models than correct models, but it does occur even in ideal cases. When the estimates did not converge in 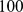 iterations, as happened in 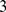 samples, we generated and used a replacement sample, so that our results are based on 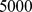 samples for which the estimates converged.

### Multiple solutions and boundary estimates: Simulation results

The frequency of samples with interior and different kinds of boundary estimates obtained in our first simulation study (under an ideal setting) is shown in Table 1. Here we have treated fitted values greater than 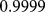 as one, and less than 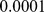 as zero. We have the following results:

**Table 1:** Counts of different kinds of fits for detection and occupancy from the simulation fitting occupancy and detection models in an ideal situation.

		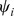						
		All 0	Some 0	Some 0 and 1	Some 1	All 1	Interior	Total
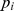	All 0	0	0	0	0	0	0	0
	Some 0	0	0	0	0	0	0	0
	Some 0 and 1	0	0	9	0	0	0	9
	Some 1	48	1	11	0	0	0	60
	All 1	62	0	0	0	0	0	62
	Interior	10	0	21	57	12	4769	4869
	Total	120	1	41	57	12	4769	5000

The true values are 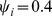 (or 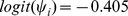) and 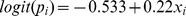.

- **vglm** produces interior estimates for both 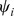 and 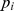 in 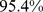 of samples and boundary estimates of various kinds in the remaining 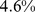 of samples. This shows that, even in the present ideal setting, the sampling variability of the estimates is very large.
- There are 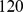 (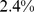) samples with all 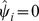 and nonzero 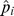. This seems strange because, if there is nothing to detect, the detection probability is zero. In fact, these samples do have a few detections (so there are definitely occupied sites) but tend to have a non-monotonic pattern in the number of detections as the covariate increases. This implies that we should interpret 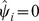 as meaning the occupancy probability is small rather than literally zero.
- The samples with 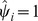 also tend to be sparse and/or to have a non-monotonic pattern in the number of detections as the covariate increases. Even though the data patterns are similar, the estimates are on the opposite boundary because small changes in sparse data have large effects on the estimates (see below).

- For samples with 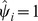, even after detailed and careful searching, it is not easy to tell whether there are other solutions with 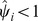. We refitted the model to these samples with different starting values but each time found only the boundary solution with all 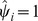. In this situation, it makes sense to study the estimates returned by **vglm** under its default settings. Having said this, **vglm** is good at finding estimates with 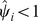 when these exist, and it usually returns the maximum likelihood estimates.

Table 1 shows that when we present and study simulation results, we have to take into account that they typically include some boundary estimates whose fitted values are meaningful but whose parameter estimates and standard errors are meaningless, as some of them should actually equal infinity or negative infinity. These estimates and standard errors swamp plots such as histograms so we cannot see any detail and make computing the means and standard deviations of the estimates and standard errors meaningless. We therefore have to exclude these extreme values from plots such as histograms to show detail and use robust trimmed estimates rather than means and standard deviations. We will point out whenever we have had to do this.

**Figure 4:**
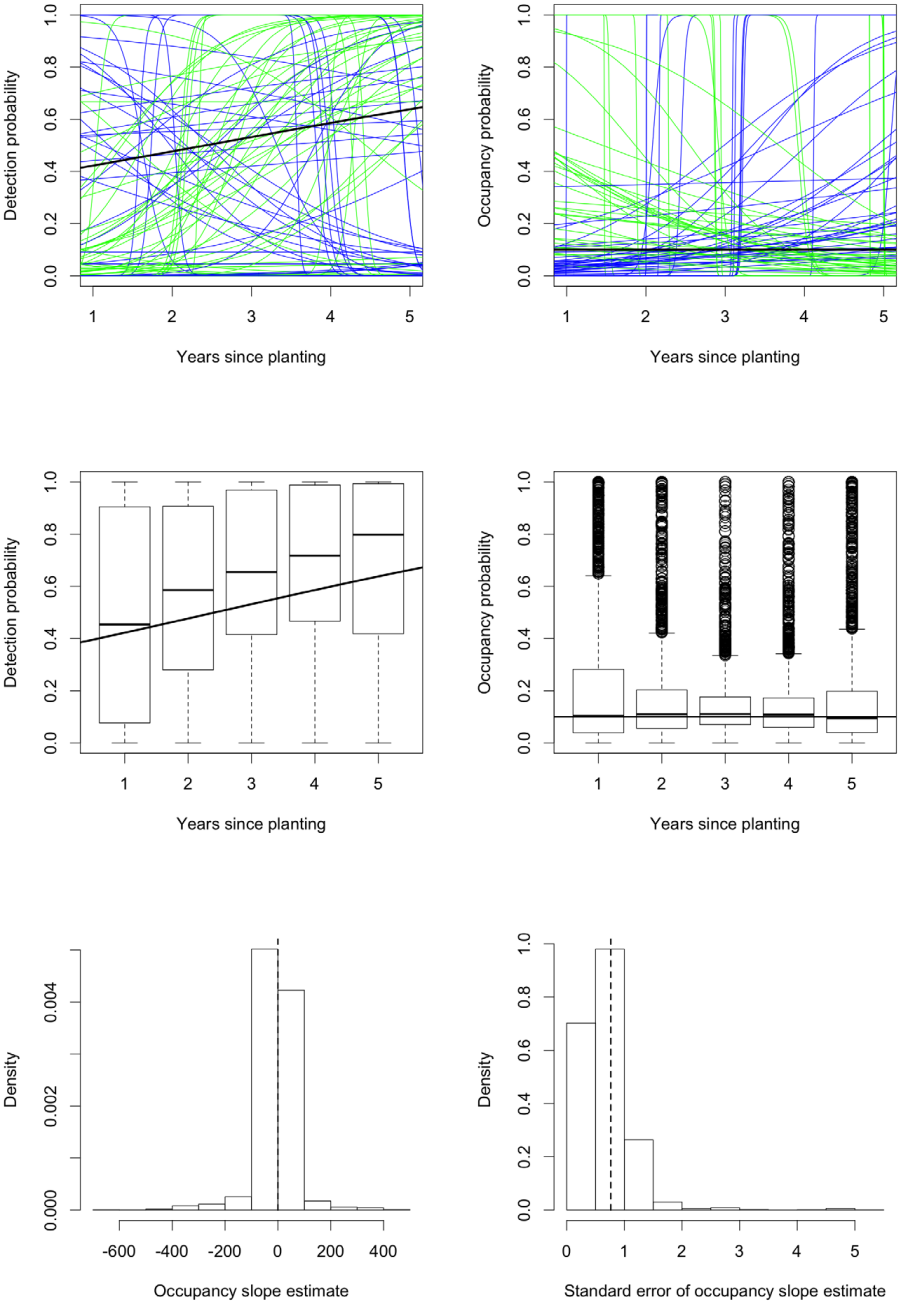
Simulation results for fitting occupancy models in an ideal situation. The first row shows fitted logistic curves for detection and occupancy for the first 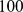 samples. The samples with positive/negative fitted relationships between occupancy and years since planting the surrounding Radiata pine are shown in blue/green; the true relationship is shown in black. The middle row shows boxplots of the fitted values for detection and occupancy for each year since planting; the true relationship is again shown as a black curve. The final row shows histograms (after trimming 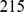 values with standard error greater than 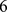) of the estimates of the slope in the occupancy component of the model and the standard errors of these slopes; the vertical dashed lines are the true value of the slope parameter and the standard deviation of the slope estimates.

Figure 4 shows several graphical presentations of the simulation results from **vglm**. The first row shows the fitted logistic curves for occupancy 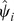 and detection 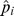 as functions of the years since planting 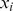 for the first 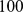 simulated samples. In both panels, the samples which produce a positive/negative estimated relationship between occupancy and years since planting are shown in blue/green while the true relationship is shown in black. (In the simulations, the true occupancy probability is always constant, equal to 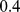 or 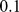, and the true detection probability depends on the simulation.) There are approximately equal numbers of increasing and decreasing occupancy curves; out of 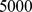 samples, 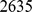 (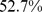) have negative occupancy slope estimates. The figures are too dense to see anything if we plot all 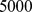 curves, so instead, in the middle row of Figure 4, we use boxplots to present the distributions of the fitted values 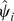 and 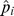 for each 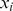 from all 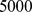 simulations. These tell the same story as the first 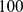 curves; the fitted values are symmetrically distributed around the true values. The variability in these plots is very large, with the distributions covering most of the possible range. The variability can be made smaller by increasing the sample size; the point here is that for our real data from the Nanangroe Study, we do not obtain very precise estimates. The final row in Figure 4 presents histograms (after trimming 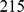 (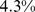) values with standard error greater than 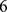) of the sampling distributions of the estimates of the slope parameter in the logistic model for occupancy and the standard errors of these slope estimates. The sampling distribution of the slope estimates is symmetric about zero. The sampling distribution of the standard errors is asymmetric with a long right tail so the standard errors are often slightly smaller than the value they should be, namely the standard deviation of the estimates across simulations. Figure 4 shows that in ideal situations vglm works well.

We also compared the distribution of the estimates 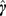 when the occupancies 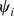 are unconstrained and when they are set to one (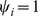) by plotting scatterplots of these estimates under the two cases. These scatterplots are not included here, but letting 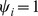 makes the estimates of 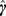 negatively biased (as we are doing a simulation, we know the true values) and much less variable than when 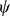 is estimated. We will see this combination of increased 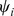 and decreased 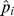 in other situations.

### Sparse data: Simulation

To simulate sparse data and investigate its effect on occupancy models, we carried out a second simulation using the setting described above but with the occupancy probability reduced to 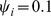. This value of the occupancy probability produces very sparse data in which we expect only 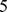
–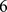 sites to be occupied. We actually observe cases like this in our Yellow-rumped Thornbill data so it is a realistic case. We can also obtain sparse data by reducing the detection probabilities; the effect is similar to reducing occupancy so we only report the results for reduced occupancy probability.

### Sparse data: Simulation results

**Figure 5:**
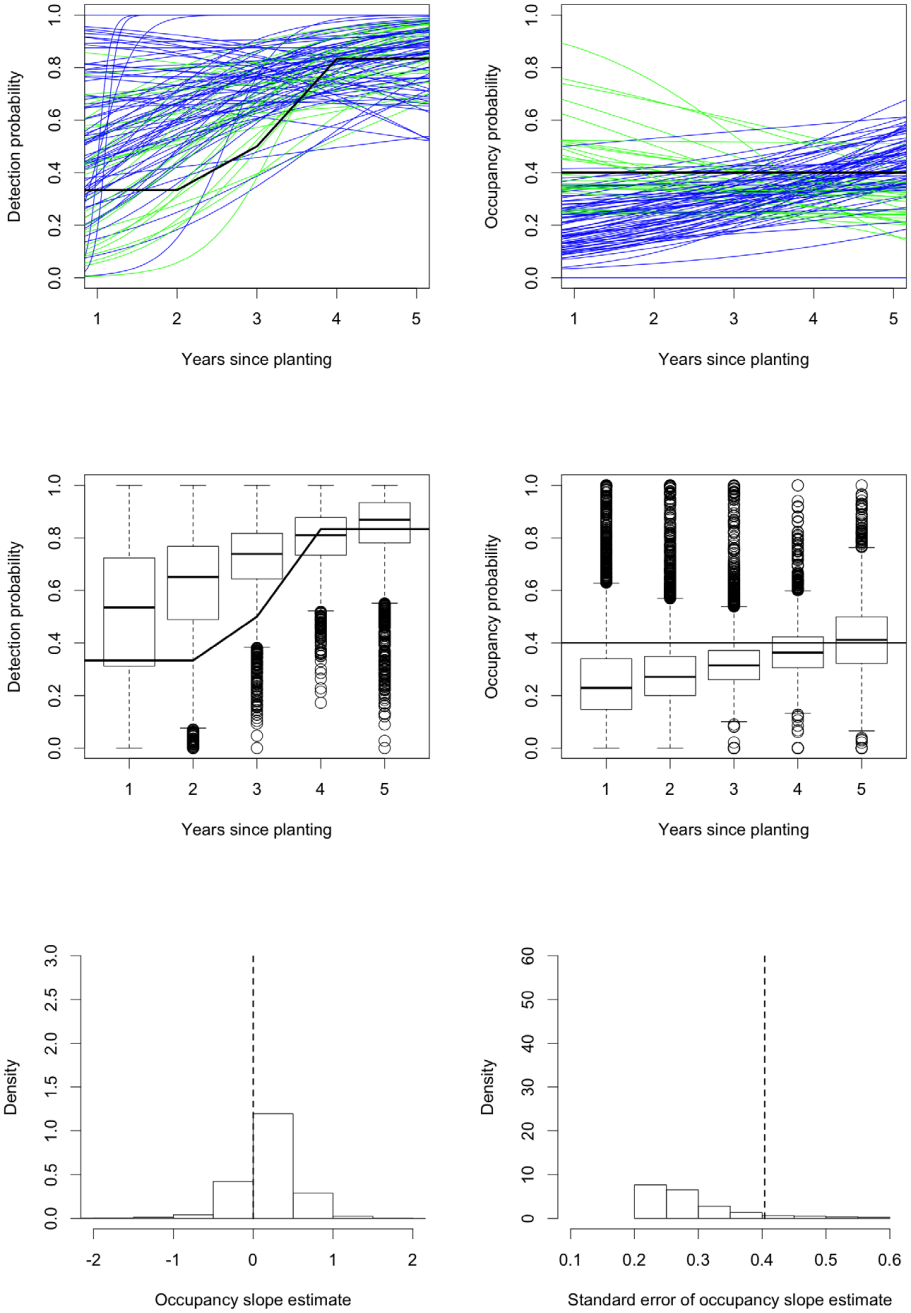
Simulation results for fitting occupancy models to sparse data. The first row shows fitted logistic curves for detection and occupancy for the first 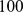 samples. The samples with positive/negative fitted relationships between occupancy and years since planting the surrounding Radiata pine are shown in blue/green; the true relationship is shown in black. The middle row shows boxplots of the fitted values for detection and occupancy for each year since planting; the true relationship is again shown as a black curve. The final row shows histograms (after trimming 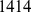 values) of the estimates of the slope in the occupancy component of the model and the standard errors of these slopes; the vertical dashed lines are the true value of the slope parameter and the standard deviation of the slope estimates.

The frequency of samples with normal and different kinds of abnormal convergence obtained in our second simulation study (of sparse data) is shown in Table 2. In contrast to the ideal case shown in Table 1, **vglm** produces interior estimates for both 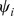 and 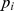 in only 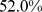 of samples and boundary estimates of various kinds in the remaining 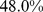 of samples. This increased tendency to estimate extreme values for both 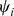 and 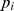 is shown graphically in Figure 5. There is both an increase in the variability in the fitted detection probabilities, as well as a positive bias for each cohort (i.e., each value of years since planting). The fitted occupancy probabilities are unbiased for each cohort but are much more variable than before. The greater variability in the estimates is reflected in the fact that 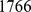 (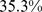) occupancy slope estimates have an estimated standard deviation greater than 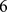 compared with 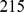 (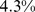) when 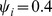. The key point is that we obtain many more extreme fits for both detection and occupancy. Intuitively, with sparse data, small changes have large effects, resulting in more extreme fits. Thus, data sparsity may be an explanation for the Yellow-rumped Thornbill results after 2001 (Figure 3), where it is plausible that occupancy is low and detection should be decreasing.

**Table 2:** Counts of different kinds of fits for detection and occupancy from the simulation fitting occupancy and detection models when the data are sparse.

		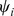						
		All 0	Some 0	Some 0 and 1	Some 1	All 1	Interior	Total
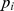	All 0	0	0	0	0	0	0	0
	Some 0	0	0	58	77	24	5	164
	Some 0 and 1	0	40	282	0	0	420	742
	Some 1	36	4	75	0	0	147	262
	All 1	242	0	0	0	0	58	300
	Interior	70	42	523	98	202	2597	3532
	Total	348	86	938	175	226	3227	5000

The true values are 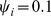 (or 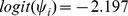) and 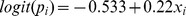.

### Detection a function of abundance: Simulation

To explore the effect of underlying abundance, we suppose that 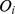 follows the model (1) and we then separately model the abundance (or, more precisely, the site-specific conditional abundance) 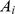 for each occupied site. We set 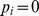 if 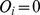 and 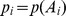 otherwise to generate the data, and then treat the observed detection data 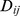 as being generated by the model (2). We cannot observe the abundance 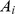 so our modelling is still to fit the occupancy model (1)–(3) to these data. The model we fit to the data is not the same as the model we used to generate the data so the question we want to address is what is the impact of fitting the occupancy model (1)–(3) when detection is actually a function of abundance?

We carried out a simulation using the setting based on the Nanangroe Study described above. We set 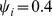 so 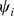 is constant and does not depend on abundance 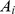 or years since planting 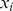. We generated the nonzero detection probabilities for 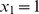 and 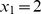 from the beta(0.5,1) distribution, for 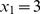 from the beta(1,1) distribution and for 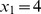 and 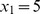 from the beta(10,2) distribution. (This makes 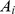 continuous but this approximation is widely used in logistic-normal models, can be avoided with slightly greater complexity by using discrete versions of the continuous distributions, and does not really affect the conclusions.) We then generated detection data from 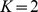 visits and fitted the occupancy model using 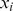 as the covariate. Our results were again based on 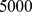 samples for which the estimates converged.

We generated the data to allow the abundance 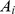 to depend on 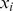. This follows because 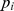 depends on 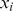 and we can derive the implied value of 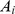 from 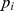; for the logistic model, 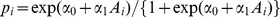, so 
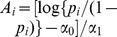
, for the model used by [10], 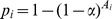, so 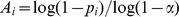, and similarly for other models. It is more convenient to generate the random 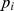 (as a function of 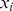) directly than to equivalently generate the 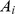 and then compute 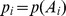.

### Detection a function of abundance: Simulation results

The frequency of samples with normal and different kinds of abnormal convergence obtained in our third simulation study (in which detection is a function of abundance) is shown in Table 3. We find that 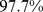 of the samples produce interior convergence estimates for both 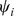 and 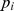.

**Table 3:** Counts of different kinds of fits for detection and occupancy from the simulation fitting occupancy and detection models when detection depends on abundance.

		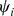						
		All 0	Some 0	Some 0 and 1	Some 1	All 1	Interior	Total
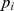	All 0	0	0	0	0	0	0	0
	Some 0	0	0	0	0	0	1	1
	Some 0 and 1	0	0	1	0	0	11	12
	Some 1	15	0	1	0	0	50	56
	All 1	20	0	0	0	0	3	23
	Interior	0	0	2	9	0	4887	4898
	Total	35	0	4	9	0	4952	5000

The true values are 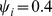 (or 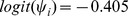) and 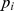 follows various beta distributions.

**Figure 6:**
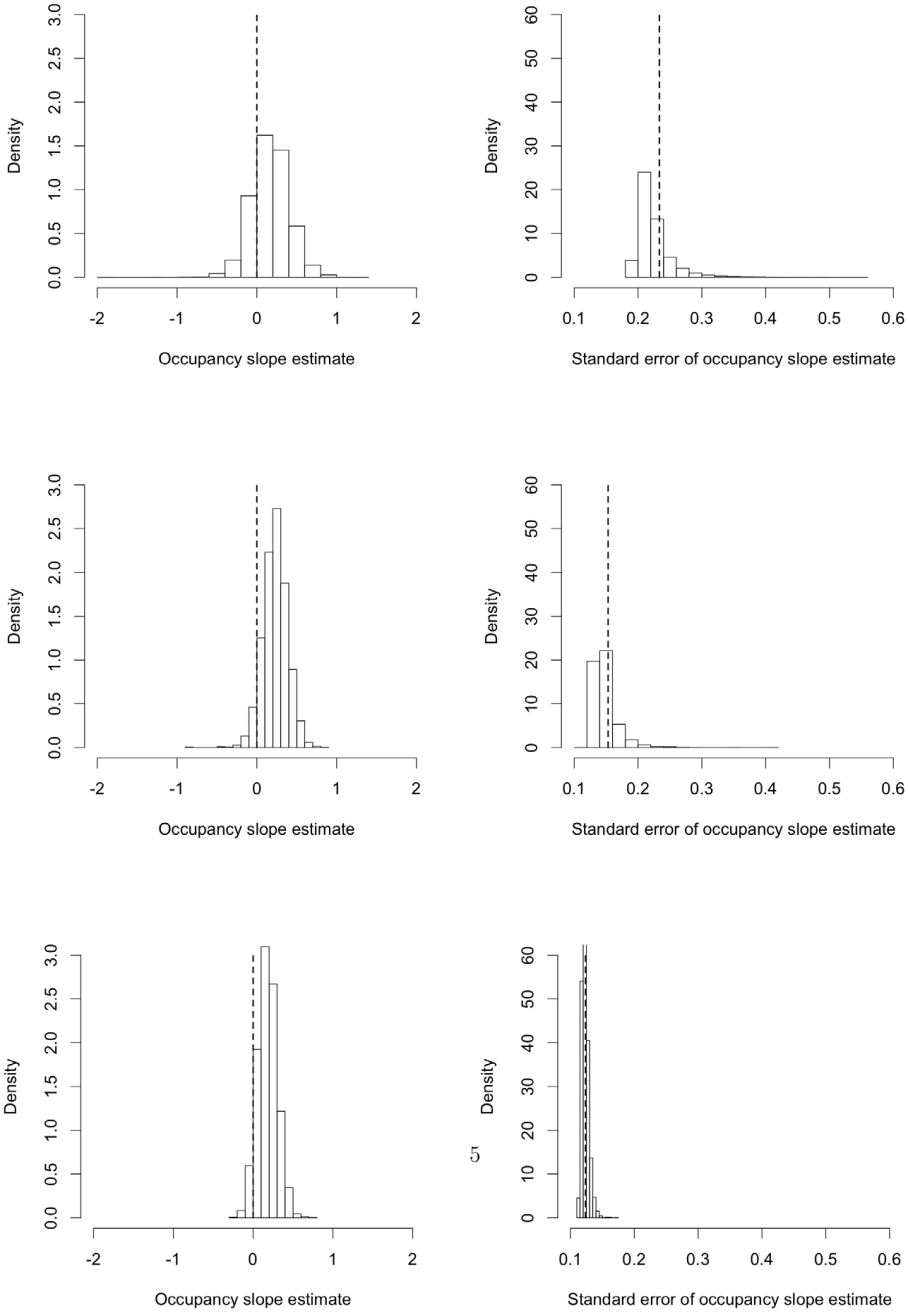
Simulation results for fitting occupancy models when detection depends on abundance. The first row shows fitted logistic curves for detection and occupancy for the first 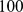 samples. The samples with positive/negative fitted relationships between occupancy and years since planting the surrounding Radiata pine are shown in blue/green; the true relationship is shown in black. The middle row shows boxplots of the fitted values for detection and occupancy for each year since planting; the true relationship is again shown as a black curve. The final row shows histograms (after trimming 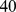 values with standard deviation great than 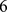) of the estimates of the slope in the occupancy component of the model and the standard errors of these slopes; the vertical dashed lines are the true value of the slope parameter and the standard deviation of the slope estimates.

The first row of Figure 6 shows the fitted logistic curves for detection 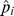 and occupancy 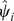 as functions of the years since planting 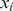 for the first 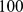 simulated samples. As in Figure 4, the samples which produce a positive/negative estimated relationship between occupancy and years since planting shown in blue/green. We find that 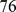 out of 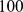 simulations show a positive estimated relationship between occupancy and years since planting, even though the true occupancy (in black) is actually constant. The plot of detection against years since planting (the first panel) shows that the simulations which produced a positive estimated relationship between occupancy and years since planting (blue) tend to have higher detection probability for low values of years since planting and lower detection probability for high values of years since planting than the simulations which produced a negative estimated relationship between occupancy and years since planting (green). We use boxplots to present the distributions of the fitted values of 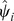 and 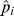 for each 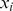 from all 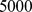 simulations in the second row of Figure 6. Again, for few years since planting, occupancy tends to be underestimated and detection overestimated and, for many years since planting, the median values of occupancy and detection are closer to the true values. The distributions cover the whole or nearly the whole range of possible values for each value of years since planting. This shows that we do not estimate occupancy and detection particularly well in this situation.

The third row in Figure 6 presents histograms of the sampling distributions of the 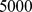 estimates of the slope parameter in the logistic occupancy component and the estimated standard errors of these slope estimates. Both histograms exclude some extreme estimates so that we can see the detail in the centre of the distributions and so we can use the same axes for later comparisons. The first histogram excludes 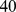 (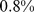) estimates with standard errors greater than 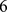; the second excludes 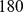 (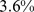) of the standard errors in the upper tail which are greater than 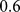. The dashed vertical line in the histogram for the slope estimates is at the true value of the slope parameter (i.e., zero); the dashed vertical line in the histogram for their standard errors is at the standard deviation of the slope estimates over the 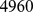 simulation estimates. The histogram for the slope estimates shows that most of the estimates are positive (with 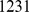 negative estimates, zero is at the 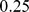 quantile of the distribution). In addition, the distribution is left skewed with a long lower tail. Although the mean of the distribution is roughly in the right location (i.e., near zero), the modal estimate is positive and we have a high probability of obtaining a positive slope estimate. The histogram for the standard errors shows a right skewed distribution with large variability. A high proportion of the standard errors are smaller than the true value.

**Figure 7:**
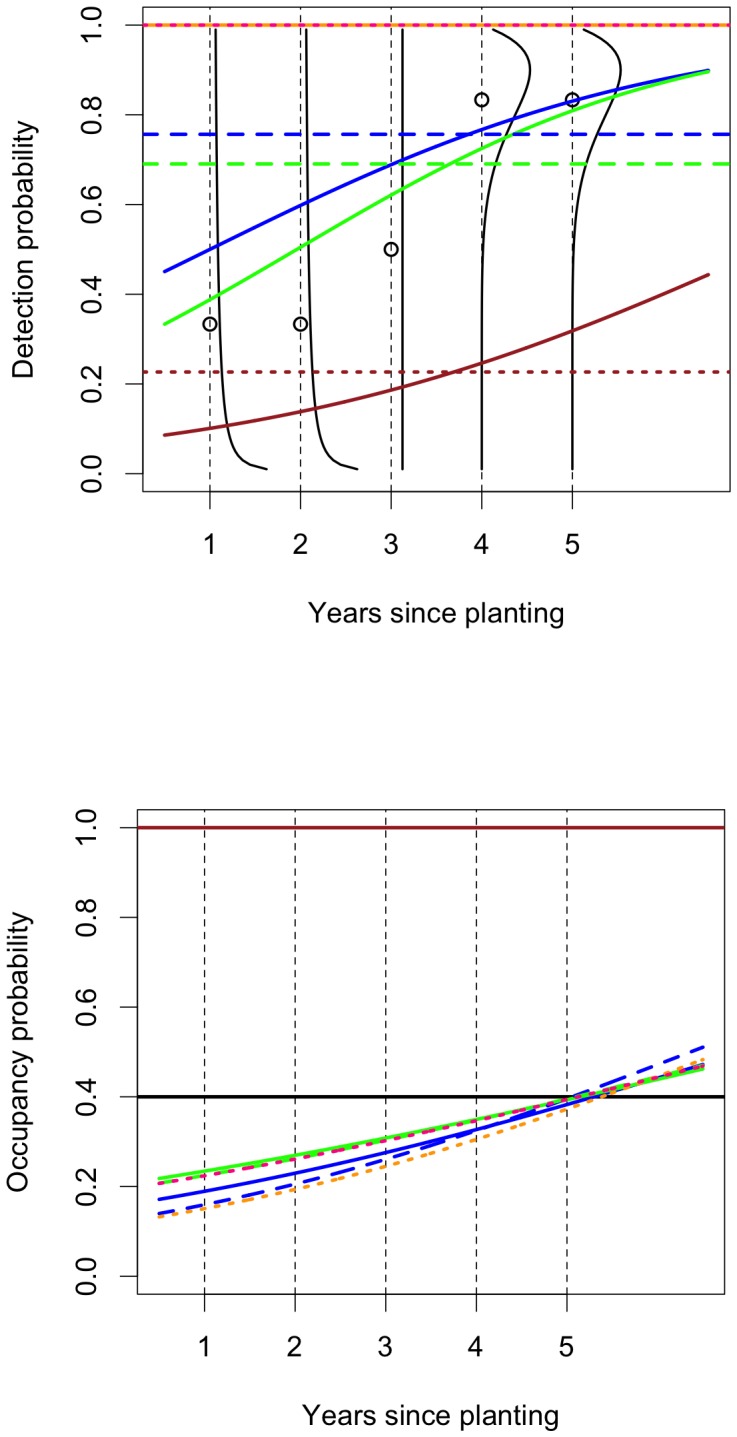
Simulated sampling distributions of the slope estimates and their standard errors when fitting occupancy models when detection depends on abundance. Histograms of the estimates of the slope in the occupancy component of the model and the standard errors of these slopes for 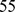 sites with 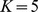 visits, 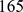 sites with 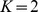 visits and 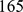 sites with 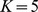 visits. The vertical dashed lines are the true value of the slope parameter and the standard deviation of the slope estimates.

Figure 7 presents the same histograms as the third row of Figure 6 but with the number of visits increased from 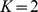 to 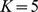 and for surveys of 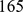 sites with 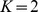 and 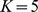 visits, respectively. No estimates have been omitted from the histograms. To facilitate comparison, the histograms for the slope estimates are all drawn on the same axes and the histograms for the standard errors are all drawn on the same axes. In the three situations considered, the number of negative slope estimates is 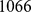 (so zero is at the 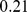 quantile), 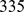 (zero is at the 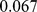 quantile) and 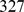 (zero is at the 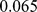 quantile). Thus, the results for the slope estimates get slightly worse as the number of visits 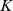 increases and much worse as the number of sites 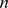 increases. For the standard errors, as we would expect, increasing 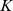 and/or 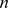 decreases the true value of the standard deviation of the slope estimates. However, the sampling distribution of the standard errors shifts location from being mostly too small, through being about right to being too large or mostly too large, showing that making inference about the occupancy slope parameter is difficult.

When we fit the occupancy model with a logistic occupancy component and constant detection instead of the occupancy model (1)–(3) to the simulation samples, we tend to get even stronger positive relationships between occupancy and years since planting. Also, there is an even greater tendency to overestimate detection for few years since planting and to underestimate detection for many years since planting with constant detection than with logistic detection.

### Detection a function of abundance: Theoretical calculations

The parameter 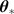 that is actually being estimated by 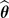 satisfies the expected estimating equations 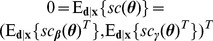, where 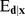 denotes the conditional expectation over 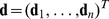 given 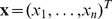; see for example [11]. We evaluate the expected estimating equations and then solve them numerically for 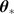. The bias in the estimates when detection is a function of abundance is given by the difference between 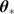 and the parameters used in the model to generate the data. In particular, for the coefficient 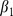 of the years since planting in the model (1)–(3), in the simulation setting the value used in the model to generate the data is zero so the bias is just 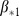, the corresponding component in 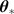. We computed 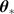 for different fitted models and different values of the number of visits 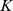 to investigate the effect of these choices on bias.

In addition to the bias in the maximum likelihood estimates 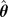, we also investigated the effect of detection being a function of abundance on the standard errors of the estimates. When detection is a function of abundance, in large samples, the standard errors should be estimating the true standard deviation obtained by taking the square root of the diagonal terms in the matrix 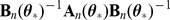, where the negative expected Hessian or Fisher information matrix 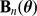 and the conditional variance of the score function 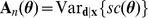 are given in the Information S1; see for example [11]. When we ignore the fact that detection is a function of abundance, the standard errors are estimating the square root of the diagonal terms of 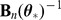. We computed both of these quantities for different fitted models and different values of the number of visits 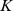 to investigate the effect of these choices on the standard errors.

In both sets of calculations, we also included cases with 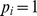 to show what happens when we ignore the possibility of non-detection and simply model occupancy directly.

### Detection a function of abundance: Theoretical results

Let the true occupancy probability given 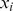 be 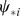 and define
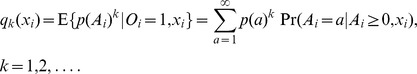
Then we can write
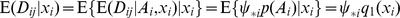
and, for 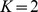,
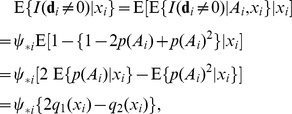
while, for 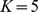,
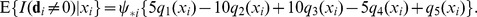
We can calculate the probabilities 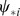 and 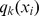 for the process actually generating the data and then solve the expected estimating equations with these values substituted into the expected score functions. These equations also have multiple solutions; the solution being estimated by the maximum likelihood estimates is the one that maximizes the the expected log-likelihood 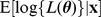.

In the setting for the third simulation, we generated the data with 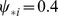 so this is already known. When we generate the 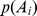 from a continuous beta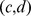 distribution with density 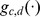, 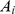 has density 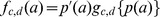, so
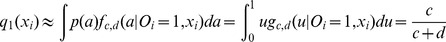
and, similarly,
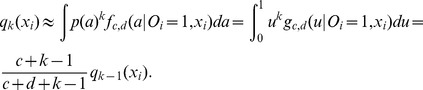
Substituting these values into the expected estimating equations and solving, we obtain the results shown in Table 4. The occupancy parameter estimates are biased and this bias does not vanish in large samples. Under the logistic occupancy model, the occupancy slope parameter is positively biased so we estimate positive relationships between occupancy and 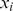 even though there is no relationship between occupancy and 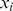. Under the constant occupancy model, we underestimate occupancy. The four rows with 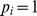 are particularly important because these show the biases we obtain when we ignore the possibility of non-detection and simply model occupancy directly. These biases are very similar to the others in the table, showing that there is no gain in fitting the occupancy model over ignoring the detection process entirely.

**Table 4:** Solutions of the expected estimating equations that maximize the expected log-likelihood under the settings used in the simulations.

Occupancy	Detection	Visits	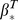	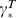
logistic	logistic	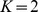	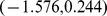	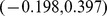
logistic	logistic	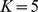	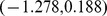	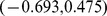
logistic	constant	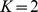	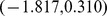	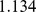
logistic	constant	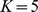	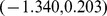	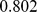
logistic	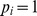	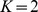	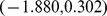	
logistic	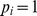	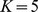	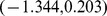	
constant	logistic	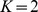	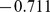	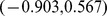
constant	logistic	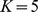	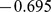	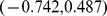
constant	constant	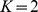	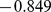	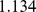
constant	constant	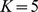	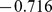	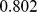
constant	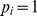	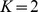	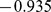	
constant	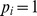	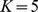	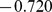	

The true value for 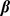 in all cases is 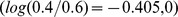.

The first six of the models in Table 4 (all with logistic occupancy components) are illustrated in Figure 1. (We cannot show all the models because the fitted lines are physically on top of each other.) The constant detection lines show greater attenuation (i.e., they are too high for small 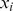 and too low for large 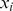) but there is considerable attenuation in the logistic detection curves too. For the 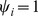 solution, the detection curves are much lower than they should be. The detection curves at 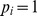 (i.e. ignoring non-detection) are too high. The corresponding occupancy curves are plotted in the second panel with the same pattern and colour combinations. The logistic occupancy curves all show an increasing relationship between occupancy and years since planting. The occupancy curves obtained when we ignore the possibility of non-detection are very similar to the curves obtained when we fit occupancy models. Table 4 also shows that if we fit the simpler constant occupancy probability, the bias changes, but does not go away.

The standard deviations of the occupancy and detection slope estimates in the simulation settings are presented in Table 5 for 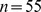; the values for 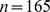 are obtained by multiplying those in the table by 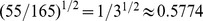. The standard deviations for 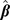 are not much affected by the fact that detection is a function of abundance in the settings we consider. (They may be in other settings but that is beyond the scope of this paper.) On the other hand, some of the standard deviations for 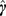 which ignore the fact that detection is a function of abundance are smaller than they should be, making these inferences about 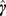 over optimistic.

**Table 5:** The standard deviations of the parameter estimates under the settings used in the simulations.

Occupancy	Detection	Visits	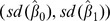		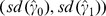	
			ignore	true	ignore	true
logistic	logistic	*K* = 2	(0.903,0.246)	(0.901,0.245)	(1.395,0.390)	(1.419,0.394)
logistic	logistic	*K* = 5	(0.724,0.210)	(0.723,0.209)	(0.609,0.183)	(0.838,0.236)
logistic	constant	*K* = 2	(0.800,0.227)	(0.814,0.227)	0.520	0.520
logistic	constant	*K* = 5	(0.712,0.207)	(0.713,0.207)	0.231	0.317
logistic	*P*_i_ = 1	*K* = 2	(0.820,0.233)	(0.845,0.239)		
logistic	*P*_i_ = 1	*K* = 5	(0.712,0.207)	(0.712,0.207)		
constant	logistic	*K* = 2	0.366	0.391	(1.332,0.369)	(1.700,0.440)
constant	logistic	*K* = 5	0.291	0.292	(0.628,0.187)	(0.900,0.250)
constant	constant	*K* = 2	0.315	0.315	0.520	0.520
constant	constant	*K* = 5	0.288	0.288	0.231	0.317
constant	*P*_i_ = 1	*K* = 2	0.315	0.322		
constant	*P*_i_ = 1	*K* = 5	0.288	0.288		

The label ‘ignore’ denotes the square root of the diagonal elements of 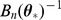 which is what we estimate when we ignore the measurement error, and ‘true’ denotes the square root of the diagonal elements of 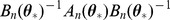 which is what we estimate when we take the measurement error into account.

### Ignoring possible non-detection: Simulation

To further explore the effect of ignoring the possibility of non-detection when we model occupancy, we fitted ordinary logistic regression models relating occupancy (defined to equal one if the species was detected in either of the 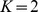 visits to a site and zero otherwise) and years since planting the surrounding Radiata pine plantation to the simulated data sets we generated above. We used the identical data we simulated to represent an ideal situation, a situation with sparse data and a situation with detection a function of abundance, so the results are directly comparable to those obtained by fitting occupancy models to these datasets.

### Ignoring possible non-detection: Simulation results

We find that in the ideal case and when detection is a function of abundance, 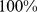 of samples produced interior convergence estimates; in the sparse data case, 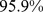 of samples produced interior convergence estimates. The boundary estimates in the sparse data simulation comprised 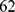 fits with all estimated occupancy probabilities equal to zero and 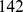 fits with some (but not all) estimated occupancy probabilities equal to zero. Comparing these values with those reported in Tables 1, 2, 3 shows that we obtain fewer boundary estimates when we ignore the possibility of non-detection.

**Figure 8:**
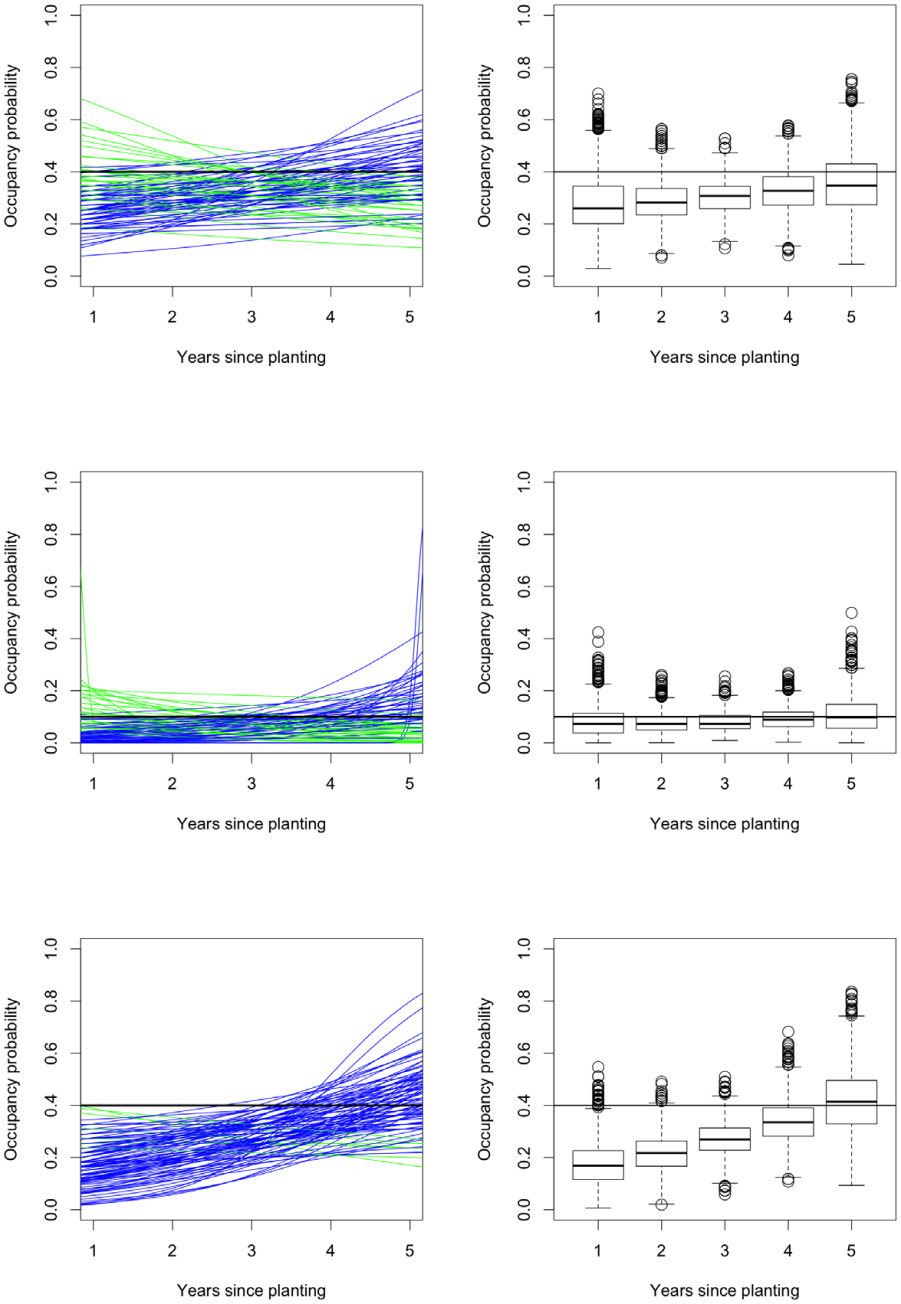
Simulation results for fitting logistic occupancy models that ignore the possibility of non-detection. The first row shows results for an ideal situation, the second for sparse data and the third for when detection depends on abundance so the rows should be compared with Figures 4, 5 and 6 respectively. The first column shows fitted logistic curves for occupancy for the first 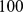 samples. The samples with positive/negative fitted relationships between occupancy and years since planting the surrounding Radiata pine are shown in blue/green; the true relationship is shown in black. The second column shows boxplots of the fitted values for occupancy for each year since planting; the true relationship is again shown as a black curve.

The simulation results are illustrated in Figure 8. The rows correspond to the three simulation settings represented by Figures 4, 5 and 6 respectively. The first column shows fitted logistic curves for occupancy for the first 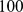 samples with positive/negative fitted relationships between occupancy and years since planting the surrounding Radiata pine plantation shown in blue/green; the true relationship is shown in black. The second column shows boxplots of the fitted values for occupancy for each year since planting the surrounding Radiata pine plantation; the true relationship is again shown in black. The distributions of fitted occupancy probabilities when we ignore the possibility of non-detection are comparable to those obtained when we fit occupancy models. The mean squared error of the estimated occupancy probability over the whole simulation (obtained by computing the mean squared error for each value of the years since planting the surrounding Radiata pine plantation and then averaging over these values) for the estimates obtained from occupancy models and for those obtained by ignoring the possibility of non-detection are 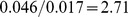 for ideal data, 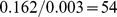 for sparse data and 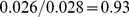 when detection is a function of abundance. Occupancy modelling is better than ignoring the possibility of non-detection in only one of the three cases and then only by a very small amount. Over the whole simulation, when we ignore the possibility of non-detection, the number of negative slope estimates is 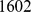 (zero is at the 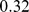 quantile) for ideal data, 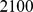 (zero is at the 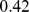 quantile) for sparse data, and 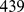 (zero is at the 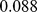 quantile) when detection depends on abundance. Thus, in the simulated data, ignoring possible non-detection lead to slightly more biased estimates of occupancy probability than occupancy modelling. However, the fitted probabilities are often less variable than those obtained by fitting occupancy models, making the mean squared errors smaller and, at least by this criterion, the estimates better.

## Discussion

Our results showing multiple and boundary solutions to the maximum likelihood estimating equations, large variability in the estimates, and the effect of sparse data mean that it is difficult to fit and interpret occupancy models. Our results on the impact of ignoring the dependence of detection on abundance have even more serious implications. Specifically, if we ignore the possibility of non-detection, the bias can be very similar and the variance smaller than if we try to adjust for non-detection. This means that ignoring non-detection can actually be better than trying to adjust for it and the extra effort of trying to adjust for non-detection is not worthwhile. Our results lead directly to the question, is it worth using occupancy models (the purported “gold standard”) at all? In this Section, we frame the problem of detection depending on abundance in the broader context of measurement error and discuss possible approaches to dealing with it.

### Measurement error

We can gain insight into the problem of detection depending on abundance, its possible consequences and the limitations of what we can try to do to deal with it by treating it as a nonlinear measurement error problem [11]. In interpreting the Nanangroe Study, a natural specification is to let the distribution of the unobserved abundance 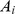 be a function of the years since planting 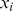, the covariate we observe and use in the model. This situation is called a Berkson model [11]. The choice we made in our simulations to have detection given 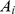 not depend on 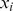, produces nondifferential error, so 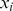 is a surrogate covariate for 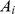 [11]. In linear models, measurement errors described by a nondifferential error Berkson model may not have much effect on the analysis, but here we have a nonlinear model so, as we have shown, more interesting effects can occur [11].

The estimating equation for 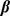 based on the score function (4) is a function of 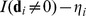, where 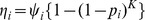. The effect of fitting an incorrect detection component in an occupancy model on the occupancy probability 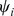 is, at least intuitively, largely captured by the ratio 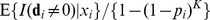. The ratio equals one and we estimate 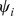 if the detection component of the model is correct and detection does not depend on abundance. The ratio is less than one so we underadjust the estimate of 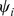 if 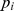 is too large, and the ratio is greater than one so we overadjust the estimate of 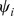 if 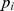 is too small. The third simulation was set up so that occupancy is underadjusted for low 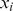 and overadjusted for large 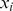. This induces the positive bias in the slope of the logistic occupancy component and the negative bias in the intercept in the constant occupancy component. We can also produce a decreasing relationship between occupancy and years since planting by letting abundance decrease with years since planting. (For example, we can switch 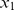 and 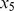, 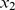 and 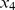.) If occupancy is also changing with years since planting, then changes in abundance can make the relationship seem stronger or weaker than it really is.

### Modelling abundance

We introduced abundance into the data generating model through a conditional, hurdle or two-part model [12] which allows the relationship between occupancy and abundance to be specified separately from the model for abundance, see [13]. A different approach is to regard abundance 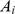 as the primary quantity and occupancy 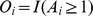 as derived from abundance. For example, if 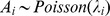, then 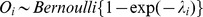, so 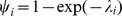. In this model, increasing the mean abundance 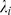 increases the occupancy probability 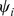 and *vice versa*, so abundance and occupancy are tied together. However, abundance and occupancy are actually quite different concepts. Intuitively, abundance is the number of individuals of a species in a patch while occupancy is the number of patches which contain at least one individual of the species. Thus, we can have changes in abundance while occupancy stays constant (the number of individuals in patches which already contain individuals changes while the number of patches containing individuals does not) or changes in occupancy while abundance stays constant (the number of patches containing individuals changes but the number of individuals in patches does not). The approach we used allows us greater flexibility in varying abundance while holding occupancy constant.

The two possible approaches to modelling abundance and occupancy shed light on the idea of [1] that we can and should replace abundance by occupancy. When we regard abundance as the primary quantity, replacing abundance by occupancy represents a sacrifice of information (simply because binary data contain less information than count data), supposedly in order to make it easier to handle detection error. Since we have shown that it is not easier to handle detection error when detection depends on abundance, this argument is not convincing. When we separate abundance from occupancy by modelling occupancy and then abundance conditional on occupancy as we have done, we see that occupancy is a different concept from abundance. A key part of ecology is the examination of both distribution and abundance [**?**] (see also [**?**]). However, occupancy is really a version of distribution rather than abundance. Therefore, we suggest that a focus on detection/occupancy modelling has the potential to detract from rather than add to the discipline of ecology.

### The Nanangroe Study

We started the present study by fitting occupancy models to data from the Nanangroe Study to relate occupancy to years since planting the Radiata pine stands surrounding the woodland patches. We found it difficult to interpret the results (reported above) at face-value. As years since planting increases, the Radiata pine plantation surrounding the patches matures and there are changes in the vegetation structure in the patches - the understorey layer becomes more dense. This kind of change may effect occupancy but it is unlikely to affect calling behaviour directly. However, according to [8] and [9], detection is expected to decrease with increasing foliage density and hence should decrease with years since planting. The Brown Thornbill and the Yellow-rumped Thornbill are members of the same guild that are detected in the same way so, if changes in the understorey layer did affect detection directly, it is unclear why they would have an general positive effect on the detection of the Brown Thornbill, in some surveys a positive effect on the detection of the Brown Thornbill and a negative effect on the detection of the Yellow-rumped Thornbill, or why detection should oscillate so wildly from survey to survey for the Yellow-rumped Thornbill. It seems more likely that the effect of changes in vegetation on detection is mediated by concurrent changes in other variables. In particular, changes in the vegetation with increasing years since planting do seem to affect the abundance of the species, causing the Brown Thornbill to increase in abundance and the Yellow-rumped Thornbill to decrease in abundance, and the abundance of the species does affect detection because it affects the probability of birds calling. Specifically, the calling behaviour of the species depends on its abundance (as well as the abundance of other species) in a patch. Our results show that changes in abundance may be inducing changes in detection and once the abundance and occupancy become low, so the data are sparse and produce wild oscillations in the fitted models.

### Survey protocol

In most studies, we expect abundance to vary across sites and for detection to depend on abundance [1]. The extent to which this is a problem depends on the method of detection, the size of the sites and the variation in abundance. The first two of these can be changed to some extent by an ecologist and it makes sense when possible to choose them to reduce abundance related problems.

It is generally good practice in a survey to try to make detection as good as possible. However, the starting point for occupancy modelling is that there are limits to how good we can make detection, so making detection perfect is not generally achievable. We are left with either choosing a detection protocol so that detection does not depend on abundance, or following the recommendation of [1] that studies should be designed so that abundance is constant over sites. Both are good solutions, when they are possible, but unfortunately, they are usually not achievable. For example, it is not possible to adjust sites when they are naturally defined islands, forest patches etc. and it is very difficult to adjust sites to achieve constant abundance of social species which form groups of different sizes, such as mixed feeding flocks which both the Brown Thornbill and the Yellow-rumped Thornbill are known to join [14]. Even if it is theoretically possible to adjust sites to achieve constant abundance for a single species, it may not be possible for multiple species simultaneously, and may require us to know more about abundance and occupancy patterns than we do. For example, we may need to know the locations and extents of home ranges or territories in different parts of a study area; having this knowledge would make it unnecessary to carry out the occupancy survey. Making abundance constant over sites is not generally possible. In any case, it is not applicable to investigations like the Nanangroe Study because the patches are the natural sampling units to which the questions of interest apply (so we cannot adjust their size) and the study is simultaneously collecting data on a large number of bird, small mammal, reptile and other species.

### We cannot observe abundance

If detection is perfect (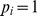), we can observe the actual occupancy 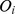 of a site by a species and it is straightforward to model the occupancy probability 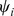 (using logistic regression rather than occupancy models). In such cases, it is more difficult but it may also (at least theoretically) be possible to observe all the individuals of a species on a site, and hence calculate the abundance 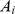. However, in occupancy modelling we allow the possibility of not detecting a species at a site and this implies that even if we detect the species, we may not detect all the individuals of that species on a site. That is, we cannot observe the abundance 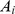. Thus, we have a circular situation; we cannot obtain the data we need to adjust correctly for non-detection but, if we could, we would have perfect detection and hence would have no need to adjust for non-detection.

### Other surrogates for abundance

The most attractive approaches for adjusting for measurement error involve trying to collect more data so that we can treat the problem empirically [11]. In our context, the measurement error is due to the unobserved abundance 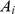 so it is natural to try to obtain some information on 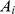 from all or some of the patches.

Although, as we have noted, we cannot observe abundance 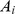, it is realistic to think about observing a version 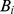 of 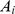 which is affected by measurement error rather than 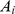 itself. In such cases, we can explore the effect of including 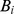 in our models. For the data collected in the Nanangroe Study, the logic behind occupancy models dictates that we must have 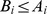, and under the assumption of no false detections, for patches with no detections we must have 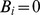. Just as observing 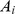 would provide us with the exact occupancy status of the patches, observing 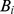 provides us with the observed occupancy of the patches. Critically, for all the patches where the species is not detected 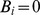, so we have a complete separation between detection and non-detection in the data with 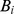 and trying to model detection as a function of 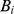 produces meaningless parameter estimates. Intuitively, observing 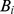 does not help with the basic issue in adjusting for non-detection, namely distinguishing between patches which are unoccupied and patches which are occupied but on which we do not detect the species, because 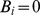 on all these patches.

The comments above apply to any attenuated version 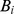 of 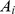 satisfying 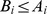. This means that in this case there is no 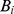 which will enable us to adjust for the measurement error which occurs when detection depends on abundance. They also apply to two-stage validation and replication studies [11], in which we observe 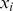 for all sites and observe 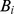 for some sites. We are left with the non-empirical solution of making assumptions about either detection or abundance. Since one objective of occupancy modelling is to estimate detection, we impose the assumptions on abundance.

In other studies of different species with different methods of detection such as methods based on counts of animal sign (so we can potentially count each animal multiple times), 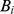 can be larger than 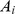. In principle, this should be helpful as it may make it plausible to assume that 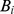 is unbiased for 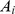 on sites for which 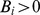. However, we still face a nonlinear measurement error problem in which the unbiasedness may not help us on the transformed scale of the response, we still have no precise measurements against which to calibrate 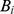, and we still have the complete separation between detection and non-detection in the data because 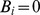 on patches with no detections.

### Latent variable approach

As we cannot observe abundance, we can include it in the detection component of the model (e.g. the logistic detection component (3)) as an unobserved missing or latent variable and then carry out a full likelihood analysis. Let 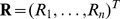 be a vector of independent latent random effects whose distribution has density function 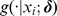, where 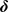 is a vector of additional unknown parameters. We use the notation 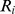 instead of 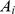 to make it easier to distinguish between the random effects we include in the model and the actual abundance which they are intended to equal, but may not. Then we include the random effects 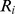 in the detection model so that the assumed detection probability becomes 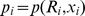. Note that this is a differential measurement error Berkson model. Replacing 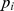 in the likelihood 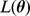 by 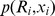 gives the conditional likelihood. Integrating the conditional likelihood over this distribution gives the likelihood for 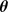 and 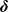 based on the observed data. The score function is 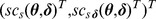, where 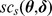 and 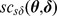 are the derivatives of the log-likelihood with respect to 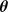 and 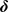 respectively. From [15], we can write 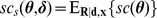 and 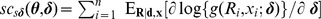, where 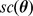 uses the assumed form for 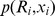 and 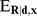, the working conditional expectation over 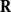 given the observed data, uses both the assumed form for 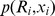 and the density function 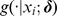. The maximum likelihood estimates are solutions of 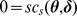 and 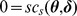. They are estimating solutions of the expected estimating equations 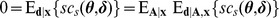 and 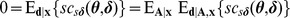. As in any model, the form we have assumed for 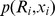 may not be correct and, in addition, the density function 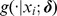 may not be the same as the density function for 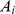. i.e. the distribution for 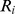 may not be the same as the distribution for 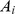. When this happens, just as we showed happens when we ignore the dependence of the detection probability on abundance, we obtain biased estimates. We can also proceed to explore the effect of misspecification on the standard errors.

The latent variable analysis is very similar to that described in chapter 5 of [1]. The main difference is that they assume that 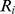 does not depend on 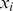 [1]. This simplification removes the problem from the measurement error framework and places it in the nonlinear mixed model framework. With nonlinear mixed models, we usually have several observations (representing a cluster of observations) with the same random effect but here we only have one observation per random effect. This raises questions about the identifiability of the model. The discussion in [1] based on [16] shows that this is a problem. They note that different models for the distribution of 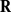 give very similar fits to the data, but different estimates of the occupancy probability. That is, the model is not identifiable but different choices give different results and hence the occupancy probability is not identifiable. Beyond acknowledging that this is a problem, advising that it should be considered and describing it as a biological sampling issue rather than a statistical issue [1], suggest applying their occupancy models anyway.

Our viewpoint differs from that of [1]. The fact that we cannot observe abundance and know little or nothing about the distribution of abundance is a fundamental problem. We are often modelling occupancy to try to avoid even greater difficulties with abundance, but whether we ignore abundance or try to include it in our analysis, abundance has not gone away and we cannot easily avoid its effects. We cannot tell empirically whether abundance is affecting our analysis or not and we cannot work out the magnitude of the effect. We cannot choose empirically between making one adjustment for non-detection and making no adjustment at all, and both can be wrong by unknown amounts. This means that, as shown in Table 4 and Figure 8, occupancy modelling with its attempt to adjust for non-detection is objectively no better than the simple analysis ignoring non-detection.

### Multistate models

Occupancy modelling is based on the idea that all sites are either occupied or not, so there is a single occupancy state. The approach can be extended to allow multiple underlying occupancy states which distinguish between occupancy by different kinds of animals (e.g. breeding or non-breeding animals) as in [1] or between occupancy at different levels of abundance as in [17], [18], [19]. The difference between the two cases is that, in the former, the response is a nominal categorical variable (i.e. represents unordered categories), while in the latter, it is an ordinal categorical variable (i.e. represents ordered categories). Just as occupancy models allow and adjust for detection error in occupancy, multistate models allow and adjust for detection and classification error in the response categories, although the number of parameters in a multistate model can increase quite rapidly with the number of states. If the categories simply represent single integers, then the multistate model is modelling abundance (although in its natural multinomial mixture form it will be highly over-parameterised); if there is only a single abundance category representing at least one detection, then the multistate model is an occupancy model. Thus multistate models lie between abundance and occupancy models but, as they typically have a small number of categories, are closer to occupancy models. If we approach multistate models as simplifying abundance models, then our motivation may be that it should be easier and hence there should be less error in trying to observe categories of abundance than in trying to observe abundance itself. However, if we approach multistate models as adding more structure to occupancy models, then it may be more difficult to observe categories of abundance than simply trying to observe occupancy.

We have not simulated data from multistate models in this paper but we can still make some comments based on our experience with the simpler occupancy models we have studied. Like occupancy models, multistate models allow and adjust for detection and classification error in the response categories and, again like occupancy models, they do not adjust for measurement error in the covariates. In particular, when detection is a function of abundance, multistate models face the same difficulties as occupancy models in adjusting for measurement error and heterogeneity. Just as with the simpler models we have explored in detail, we can envisage situations in which multistate models work well and also situations where they do not, and the practical problem is that it is so difficult to work out which kind of situation we are in.

### Where are we now?

Suppose that we are planning to collect occupancy data with a view to fitting an occupancy model and we suspect that detection is a function of abundance. We have explored in detail the approach of using a surrogate for abundance in our model and shown that this creates a nonlinear measurement error problem in which occupancy models can perform poorly. We have also discussed the approaches of including a latent abundance variable in the model and of ignoring the possibility of non-detection. All three of these approaches have unsatisfactory aspects.

The difficulties we have found and discussed are very challenging to resolve, particularly when we think detection depends on abundance. In general, the solution is not simply to construct increasingly complicated models but to try to better understand the detection process so, as we noted in our discussion of survey protocol above, we can try to improve our detection methods, find creative new detection methods, or try to find new and innovative ways of calibrating detection. New and developing technology is likely to play a key role. Improved calibration may have to rely on separate calibration studies, an admittedly daunting prospect in multi-species, multi-environment, multi-season studies requiring multiple calibration studies, although a deeper understanding of detection may reduce the burden. Even in simple situations, the main difficulty is to obtain the highly accurate measurements of true abundance required for calibration. A creative approach used by [20] and [9] involves setting up field simulations (as opposed to computer simulations) in which speakers are set up to play bird calls so that truth is known and detection probabilities can really be estimated and their relationship with call abundance explored. Of course, there are real issues with translating the artificial situation with speakers to a real situation with birds (as call abundance is not the same as bird abundance) but we may be able to minimise these as we understand the detection process better. There are no easy solutions, but solutions better than those presently available may be achievable.

In the meantime, we are not arguing that ecologists should stop using their favourite methods, but we are arguing that they should recognise the limitations of their methods, avoid imposing them on everyone else, and certainly stop pretending that they are better than they are.

## Conclusions

Non-detection is a form of informative measurement error in the response which leads to biased estimates of occupancy and potentially finding misleading relationships between occupancy and other covariates. Intuitively, ignoring non-detection in occupancy data means treating non-detection of the species at the 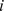
th site (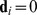) as equivalent to the site being unoccupied. The probability being modelled in this case is the raw occupancy probability 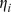. We have 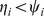 unless detection is in fact perfect (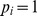). The fact that 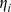 is a function of both 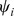 and 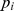 means that ignoring non-detection can induce relationships between raw occupancy and covariates that do not hold between occupancy and the covariates. This is confirmed by the 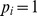 entries in Table 4 and by Figure 8. The purpose of occupancy modelling as described in [1] is to adjust the raw occupancy probability by taking into account that some of the sites at which the species is not detected are in fact occupied. This is approached by the sound empirical approach of collecting additional data (through multiple visits) rather than by simply making unverifiable assumptions about the detection process to make the problem go away.

Our analyses show that occupancy models are far more difficult to fit than is generally acknowledged. In particular, the estimating equations often have multiple solutions, making it difficult to find the maximum likelihood estimate, and there are often boundary estimates which need to be treated with more care. It would be very helpful if the models worked well when the data are sparse but the estimates are unstable in this case, making them difficult to interpret. Finally, the estimates are highly variable, making accurate inference difficult.

There is an additional source of measurement error in the data when abundance varies over sites and detection depends on abundance. Depending on how we model detection, a key covariate is either observed with unknown error, represented by another covariate (which may be a surrogate covariate following a Berkson model) or completely unobserved. When detection depends on abundance, the standard analysis suggested by [1] suffers bias (attenuation in detection, biased estimates of occupancy and potentially finding misleading relationships between occupancy and other covariates), asymmetric sampling distributions, and slow convergence of the sampling distributions to normality. Even more complicated effects are possible when there are differential errors and/or other covariates which may themselves be subject to additional measurement error. This kind of measurement error is also informative and leads to biases of similar magnitude to those we obtain when we ignore non-detection entirely.

Unless detection is perfect (i.e. we can detect every individual of a species on a site), we cannot observe abundance. If we treat abundance as a missing variable and include a latent random effect for abundance in the model, the appropriate distribution for the latent random effect is not identifiable and different distributions lead to different conclusions about occupancy [16], [1]. We can observe a version of abundance with unknown measurement error, but it is not useful to include this kind of variable in the occupancy models of [1]. This means that we have no idea of the real effect of ignoring the dependence of detection on abundance and no possibility of adjusting for this effect. We can only make assumptions which cannot be checked, and this is unsatisfactory.

Our conclusion is that occupancy modelling is more difficult than it first seems and that there is currently little we can do to obtain a meaningful analysis when detection depends on abundance. We need to better understand detection and develop new, creative ways to calibrate detection. In the meantime, it is important to be honest and realistic about what can be achieved. The problem of non-detection is a very difficult problem and difficult problems are unfortunately difficult or even impossible to solve in simple, general ways. We need to be more sanguine about claiming that a method is the “gold standard” for solving these kinds of problems.

## Supporting Information

Information S1
**Expressions for the matrices that appear in the approximate standard errors of the maximum likelihood estimate** 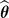 **of the parameter** 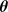 **.**
(PDF)
