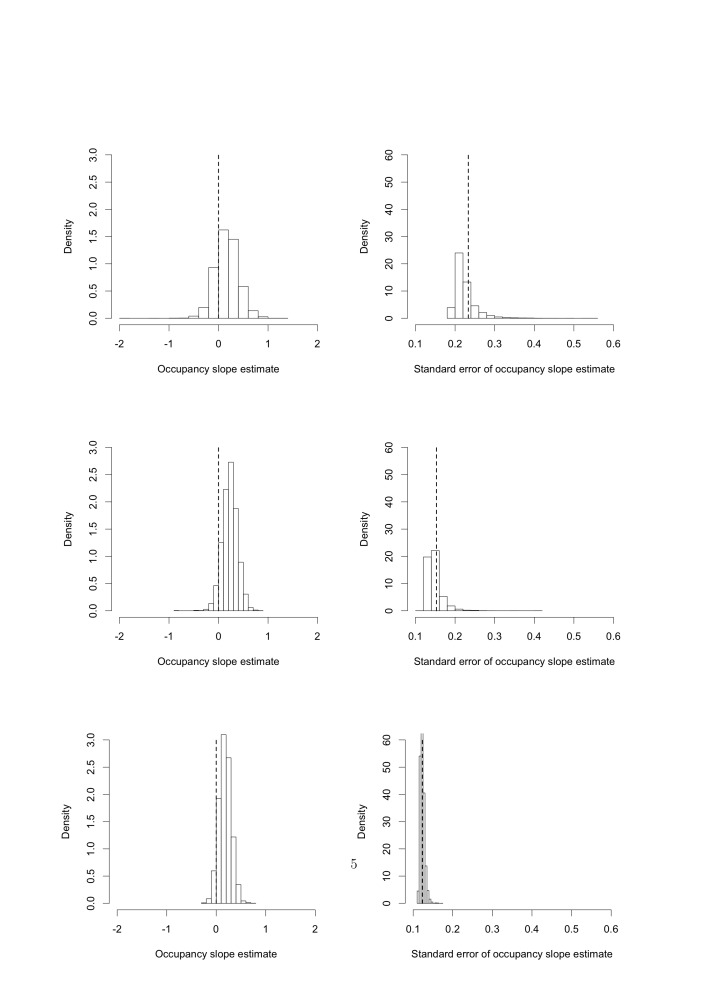# Correction: Fitting and Interpreting Occupancy Models

**DOI:** 10.1371/annotation/83cc3ff1-9438-4b1d-abf4-07f378ed558f

**Published:** 2013-04-29

**Authors:** Alan H. Welsh, David B. Lindenmayer, Christine F. Donnelly

Figure 1-7 were placed incorrectly in this article. The legends are placed correctly.

The correct Figures are:

Figure 1: 

**Figure:**
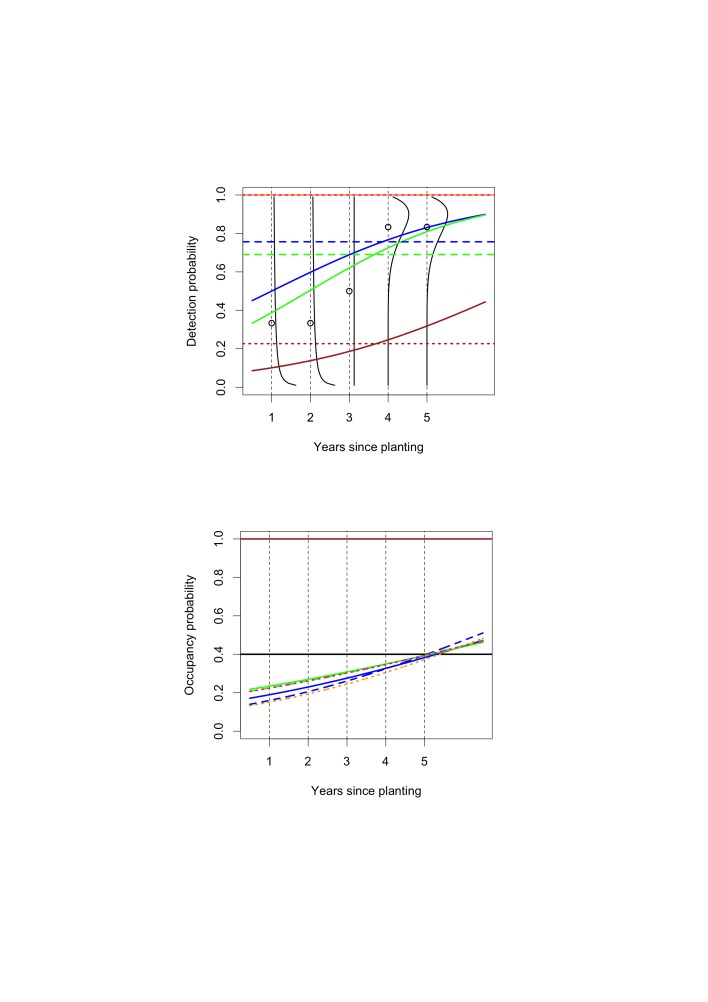


Figure 2: 

**Figure:**
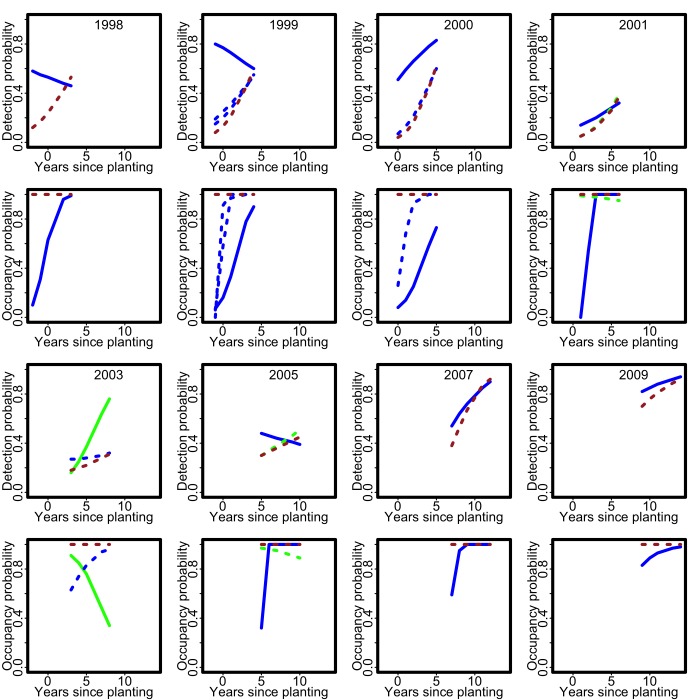


Figure 3: 

**Figure:**
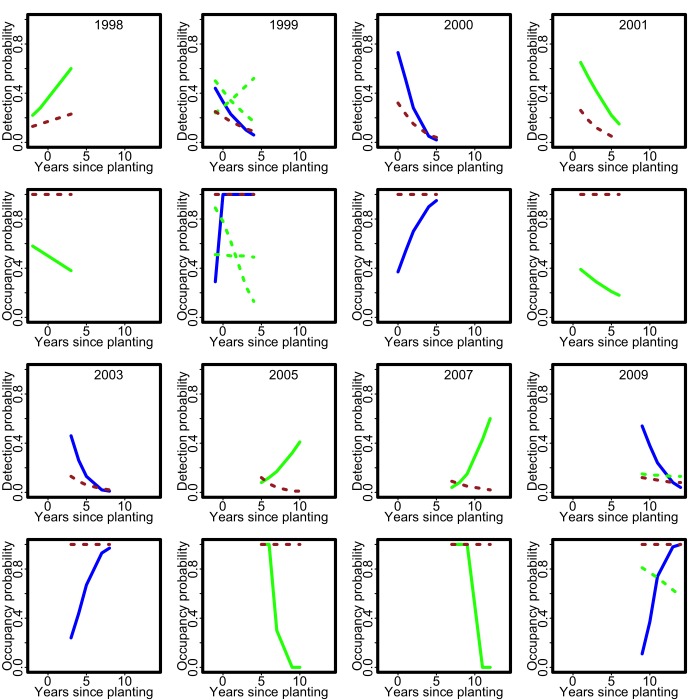


Figure 4: 

**Figure:**
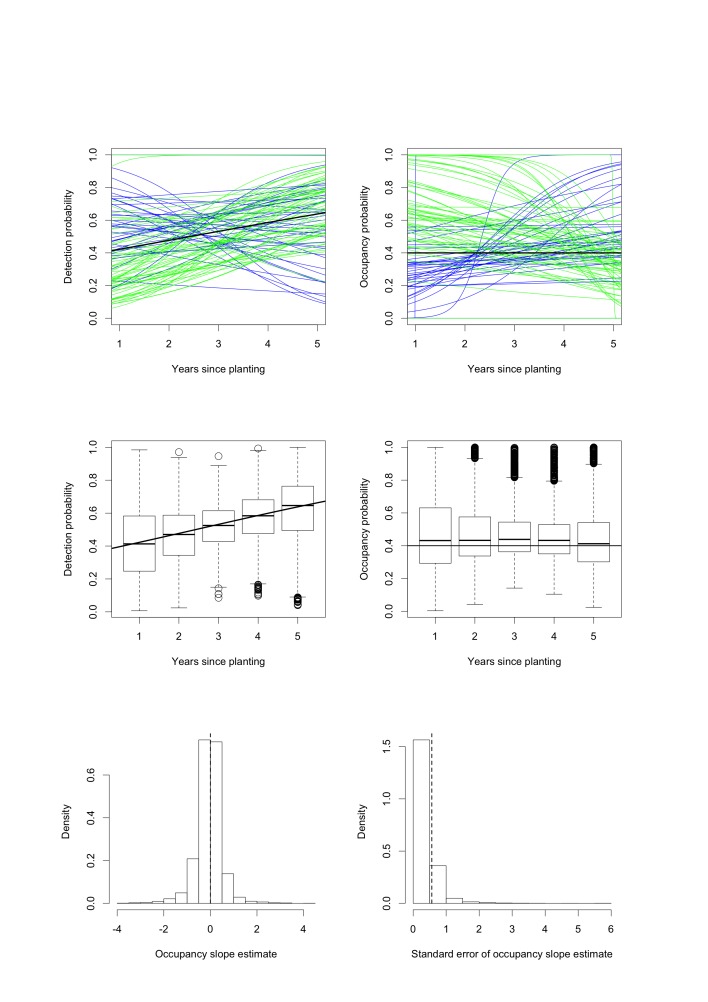


Figure 5: 

**Figure:**
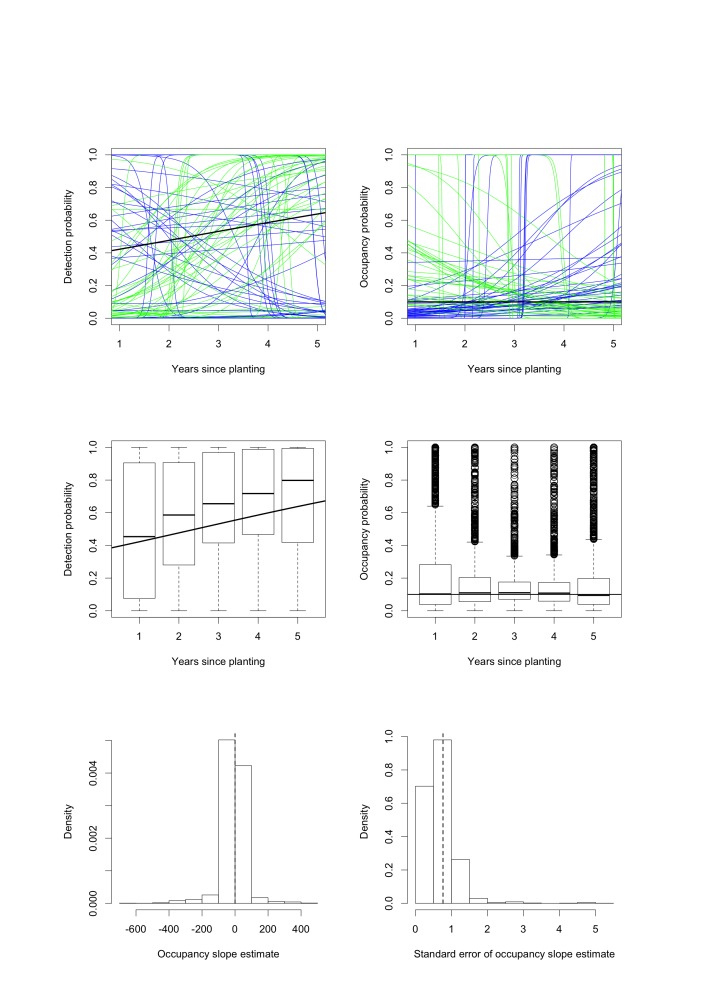


Figure 6: 

**Figure:**
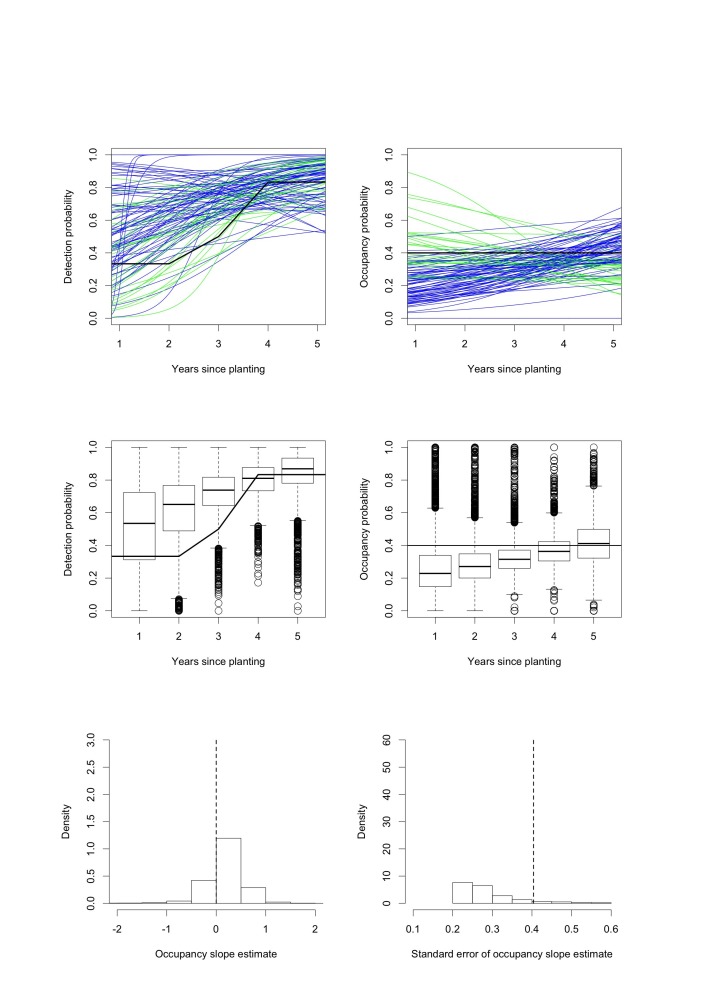


Figure 7: 

**Figure:**