# Supplementary material for: Fitting and Interpreting Occupancy Models
Source: PLoS One. 2013 Jan 10;8(1):e52015. doi: 10.1371/journal.pone.0052015 (PMC3542396; doi:10.1371/journal.pone.0052015)
Supplement: Information S1 — Expressions for the matrices that appear in the approximate standard errors of the maximum likelihood estimate of the parameter . (PDF) [file pone.0052015.s001.pdf]

Appendix

The Hessian matrix is obtained by differentiating the score functions (4) and (5) with respect to $\boldsymbol{\beta}$ and $\boldsymbol{\gamma}$. The Fisher information matrix is then minus the expected value of the Hessian matrix which, for convenience, we write in the form

$$\mathbf{B}_n(\boldsymbol{\theta}) = \begin{pmatrix} \mathbf{B}_n(\boldsymbol{\beta},\boldsymbol{\beta}) & \mathbf{B}_n(\boldsymbol{\beta},\boldsymbol{\gamma}) \\ \mathbf{B}_n(\boldsymbol{\beta},\boldsymbol{\gamma})^T & \mathbf{B}_n(\boldsymbol{\gamma},\boldsymbol{\gamma}) \end{pmatrix},$$

where

$$\begin{aligned}
\mathbf{B}_n(\boldsymbol{\beta},\boldsymbol{\beta}) &= \sum_{i=1}^{N} \mathbf{r}_i\mathbf{r}_i^T \Big(\frac{1-\psi_i}{1-\eta_i}\Big)\Big(\eta_i(1-\psi_i) + \Big(\frac{\psi_i-\eta_i}{1-\eta_i}\Big)[\mathrm{E}\{I(\mathbf{d}_i \neq \mathbf{0})|x_i\} - \eta_i]\Big), \\
\mathbf{B}_n(\boldsymbol{\beta},\boldsymbol{\gamma}) &= K\sum_{i=1}^{N} \mathbf{r}_i\mathbf{s}_i^T p_i \frac{(1-\psi_i)(\psi_i-\eta_i)}{(1-\eta_i)^2}[1-\mathrm{E}\{I(\mathbf{d}_i \neq \mathbf{0})|x_i\}], \\
\mathbf{B}_n(\boldsymbol{\gamma},\boldsymbol{\gamma}) &= K\sum_{i=1}^{N} \mathbf{s}_i\mathbf{s}_i^T p_i\Big(1-p_i-\Big(\frac{1-\psi_i}{1-\eta_i}\Big)\Big\{1-p_i+Kp_i\Big(\frac{\psi_i-\eta_i}{1-\eta_i}\Big)\Big\}[1-\mathrm{E}\{I(\mathbf{d}_i \neq \mathbf{0})|x_i\}]\Big).
\end{aligned}$$

When detection depends on abundance, the expectation is that derived Detection a function of abundance: Theoretical results. When detection does not depend on abundance, the expectation is $\mathrm{E}\{I(\mathbf{d}_i \neq \mathbf{0})|x_i\} = \eta_i = \psi_i\{1-(1-p_i)^K\}$.

To compute $\mathbf{A}_n(\boldsymbol{\theta}) = \mathrm{Var}\{\mathrm{sc}(\boldsymbol{\theta})|\mathbf{x}\}$, note that the product $(\sum_{j=1}^K D_{ij})\{1-I(\mathbf{d}_i \neq \mathbf{0})\} = (\sum_{j=1}^K D_{ij})I(\mathbf{d}_i = \mathbf{0}) = 0$, because one factor is always equal to zero. It follows that

$$\mathrm{E}\Big\{\sum_{j=1}^{K} D_{ij}I(\mathbf{d}_{si} \neq \mathbf{0})|\mathbf{x}\Big\} = K\mathrm{E}(D_{ij}|x_i) = K\psi_0 q_1(x_i).$$

Also,

$$\begin{aligned}
\mathrm{E}\Big\{(\sum_{j=1}^{K} D_{ij})^2|x_i\Big\} &= \mathrm{E}\Big[\mathrm{E}\Big\{(\sum_{j=1}^{K} D_{ij})^2|O_i=1,A_i,x_i\Big\}|x_i\Big] \\
&= \mathrm{E}\Big[\mathrm{E}\Big\{\sum_{j=1}^{K} D_{ij}|O_i=1,A_i,x_i\Big\} + \mathrm{E}\Big\{\sum_{j\neq k}^{K} D_{ij}D_{ik}|O_i=1,A_i,x_i\Big\}|x_i\Big] \\
&= \mathrm{E}\Big\{K\mathrm{E}(D_{ij}|O_i,A_i,x_i) + K(K-1)\mathrm{E}(D_{ij}|O_i,A_i,x_i)\mathrm{E}(D_{ik}|O_i,A_i,x_i)|x_i\Big\} \\
&= \mathrm{E}\{Kp(A_i) + K(K-1)p(A_i)^2|x_i\} \\
&= K\psi_0 q_1(x_i) + K(K-1)\psi_0 q_2(x_i),
\end{aligned}$$

so the matrix $\mathbf{A}_n(\boldsymbol{\theta})$ has

$$
\begin{aligned}
\mathbf{A}(\boldsymbol{\beta},\boldsymbol{\beta}) &= \sum_{i=1}^{n}\mathbf{r}_i\mathbf{r}_i^T\Big(\frac{1-\psi_i}{1-\eta_i}\Big)^2\Big[\mathrm{E}\{I(\mathbf{d}_i\neq\mathbf{0})|x_i\}-2\eta_i\mathrm{E}\{I(\mathbf{d}_i\neq\mathbf{0})|x_i\}+\eta_i^2\Big],\\
\mathbf{A}(\boldsymbol{\beta},\boldsymbol{\gamma}) &= K\sum_{i=1}^{n}\mathbf{r}_i\mathbf{s}_i^T\Big(\frac{1-\psi_i}{1-\eta_i}\Big)\Big((1-\eta_i)\mathrm{E}(D_{ij}|x_i)-p_i\eta_i\Big(\frac{1-\psi_i}{1-\eta_i}\Big)[1-\mathrm{E}\{I(\mathbf{d}_i\neq\mathbf{0})|x_i\}]\\
&\quad -p_i[\mathrm{E}\{I(\mathbf{d}_i\neq\mathbf{0})|x_i\}-\eta_i]\Big),\\
\mathbf{A}(\boldsymbol{\gamma},\boldsymbol{\gamma}) &= \sum_{i=1}^{n}\mathbf{s}_i\mathbf{s}_i^T\Big(\mathrm{E}\{(\sum_{j=1}^{K}D_{ij})^2|x_i\}-2K^2p_i\mathrm{E}(D_{ij}|x_i)+K^2p_i^2\Big(\frac{1-\psi_i}{1-\eta_i}\Big)^2[1-\mathrm{E}\{I(\mathbf{d}_i\neq\mathbf{0})|x_i\}]\\
&\quad -2K^2p_i^2\Big(\frac{1-\psi_i}{1-\eta_i}\Big)[1-\mathrm{E}\{I(\mathbf{d}_i\neq\mathbf{0})|x_i\}]+K^2p_i^2\Big).
\end{aligned}
$$
